# Synthesis and Characterization of Cholesterol-Based Liquid Crystals Linked with Perfluorinated Alkyl Chains

**DOI:** 10.3390/molecules30183731

**Published:** 2025-09-13

**Authors:** Austin Che, Carson O. Zellmann-Parrotta, Homayoun Ghaseminezhad, Jessica Duong, Vance E. Williams, Chang-Chun Ling

**Affiliations:** 1 Department of Chemistry, University of Calgary, 2500 University Drive NW, Calgary, AB T2N 1N4, Canada; austin.che@ucalgary.ca (A.C.);; 2 Department of Chemistry, Simon Fraser University, Burnaby, BC V5A 1S6, Canadahomayoun_ghaseminezhad@sfu.ca (H.G.)

**Keywords:** cholesterol, perfluoroacyl, synthesis, liquid crystals, smestic A mesophase

## Abstract

Two cholesterol-based liquid crystalline materials were synthesized by incorporating perfluorinated acyl chains of different lengths with the help of epichlorohydrin and copper(I)-mediated azide-alkyne 2+3 dipolar cycloaddition chemistries. These materials were characterized by differential scanning calorimetry, cross-polarized optical microscopy and powder X-ray diffraction. The compound with the longer perfluorinated chain exhibited a smectic A (SmA) phase as confirmed by XRD and POM, while the shorter-chain derivative exhibited diffraction peaks suggestive of both simple SmA* ordering as well as lamellar solid phase exhibiting multilayer ordering.

## 1. Introduction

**Figure 1:**
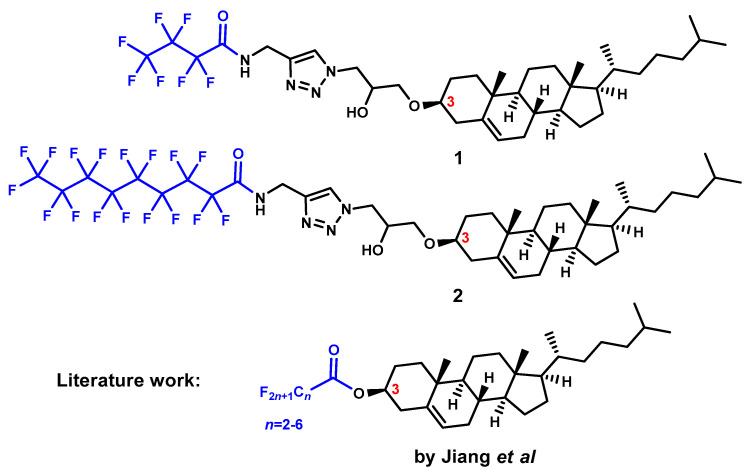
Structures of synthesized perfluorinated acyl-functionalized cholesterol targets **1** and **2** via epichlorohydrin and copper(II)-mediated azide-alkyne cycloaddition chemistry, compared to another class of perfluorinated acyl chain-modified cholesterol esters [20].

As a non-toxic, inexpensive and renewable biomolecule, cholesterol is fascinating in the liquid crystal research field because of its large rigid core structure and multi-chiral centers [1]. It is a leading mesogenic group that promotes the formation of cholesteric liquid crystalline mesophase, which is characterized by the self-organization of chiral molecules into a helical superstructure in solid states, having long-range orientational order and an absence of positional order. During recent years, cholesterol-based dimers [2,3,4,5,6,7,8,9] have attracted considerable interest because of their ability to promote the formation of multiple mesophases including chiral smectic phase (S*) [7,10,11,12,13], twist grain boundary phases (TGB*) [14], chiral nematic phase (N*) [13,15], re-entrant TGBA* phase [7,13] and blue phases (BPs) [13,16,17], and such material can exhibit unique electrical, electro-optical and optical properties. The chiral smectic phase (S*) can further exhibit ferroelectric and antiferroelectric properties [18,19,20] and materials with such properties have found important utilities in LC displays, sensors and others, because of their extremely fast operational speed and superior resolution, compared to those of the nematic technology. Acrylate- and methacrylate-functionalized cholesterol monomers have been photopolymerized to form stable chiral nematic films with tunable photonic properties [21,22]. More recently, Jiang and co-workers [20] reported a class of O3-acylated cholesterol derivatives using simple perfluorinated chains (C*_n_*F_2*n*+1_CO-) of varied length (*n* = 2–6, Figure 1), and they observed that the solid–solid phase transition temperatures of these derivatives readily increase as the number of CF_2_ groups in the perfluorinated chain grows, and the derivative with the longest perfluorinated chain (*n* = 6) shows distinct behavior as it is capable of self-assembling into cholesteric liquid crystalline mesophases. In this work, we have synthesized two novel cholesterol derivatives functionalized with perfluorinated acyl chains (C*_n_*F_2*n*+1_CO-, *n* = 3 and 8, structures **1** and **2**, Figure 1), and they are found to form smectic A mesophases. We linked the per-fluorinated acyl segment to the cholesterol via the epichlorohydrin chemistry that is further assisted with the copper(II)-mediated azide-alkyne 2+3 dipolar cycloaddition reaction (“Click” reaction) [23].

## 2. Results and Discussion

### 2.1. Chemical Synthesis and Characterization

**Scheme 1:**
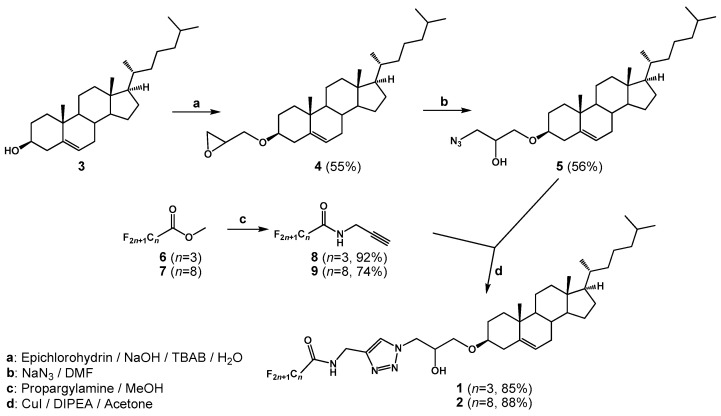
Synthesized route to the perfluorinated acyl-functionalized cholesterol targets **1** and **2**.

The target compounds **1** and **2** were synthesized according to Scheme 1. Commercial cholesterol was first alkylated with epichlorohydrin (3.0 equiv.) by following a literature procedure [24]. The reaction was carried out at 40 °C in water using sodium hydroxide (3.0 equiv.) as a base and a catalytic amount of tetra-*n*-butylammomium bromide (0.05 equiv.) as a phase-transfer catalyst. The desired cholesterol–epoxide **4** was obtained as an inseparable mixture of diastereomers by column chromatographic purification on silica gel. The obtained epoxide **4** was then stirred overnight with sodium azide (6.2 equiv.) in DMF at 80 °C; this regioselectively opened up the oxirane ring to afford the *O*-(3-azido-2-hydroxy)-substituted cholesterol derivative (**5**), also as an inseparable mixture of diastereomers. Compound **5** was isolated in 56% yield after a purification by column chromatography on silica gel using a mixture of ethyl acetate—hexanes (4:96) as the eluent. The structure of compound **5** was confirmed by ^1^H and ^13^C NMR spectra (Figures S7 and S8) that were further assisted with 2D ^1^H-^1^H GCOSY and ^1^H-^13^C GHSQC correlation experiments (Figures S9 and S10). The broad multiplet located at 3.92 ppm was determined to be the proton directly adjacent to the hydroxyl group of the 3-carbon linker, and the next two multiplets, located at 3.57 and 3.50 ppm, are assigned to be the diastereotopic methylene protons next to the ether linkage of cholesterol. Additionally, the two diastereotopic protons adjacent to the azido group are located at 3.46–3.33 ppm.

Concurrently, each of the commercially available methyl esters of perfluorinated butyrate 6 and nonanoate **7** was treated with propargylamine in methanol to afford the desired *N*-propargyl amides **8** and **9** in 92% and 74% yield, respectively. The aminolysis reaction worked well due to the high reactivity of the per-fluorinated acyl ester functionality [25], because of the strong electron-withdrawing effect of the fluorine atoms that greatly enhanced electrophilicity of the carbonyl center. Finally, each of the compounds **8** (2.0 equiv.) and **9** (2.0 equiv.) was then conjugated to the azido-functionalized cholesterol compound (**5**) under the copper (I)-catalyzed Huisgen [2+3] dipolar cycloaddition, and the reaction was carried out in acetone using *N*,*N*-diisopropylethylamine (DIPEA, 0.1 equiv.) as a base and CuI (0.1 equiv.) as a catalyst. After 12 h, the target compounds **1** and **2** were isolated in 85% and 88% yields, respectively, by column chromatography on silica gel using a mixture of ethyl acetate—hexanes (3:7) as the eluent.

1D and 2D NMR experiments were used to confirm the structures of both targets **1** (Figures S11–S15) and **2** (Figures S16–S20). For example, the successful cycloaddition between the azido (**5**) and alkyne (**9**) was evidenced by the observation of a singlet located at 7.73 ppm in each of the ^1^H NMR spectra, which correlates to the H-4 proton of the newly formed 1,2,3-triazole ring in each case. Additionally, based on 2D ^1^H-^1^H GCOSY correlation spectra, the diastereotopic methylene protons directly attached to the 1,2,3-triazole ring of both compounds **1** and **2** were observed at a much more deshielded region (4.56 and 4.43 ppm), confirming the direct attachment of the methylene groups to the 1,2,3-triazole aromatic ring. It should also be noted that the proton of the adjacent hydroxymethine group also experiences a moderate deshielding effect to 4.17 ppm, due to its proximity to the same aromatic ring. ^13^C experiments (DEPTQ) were also used to confirm the presence of the successful 3+2-dipolar cycloaddition, as the C-4 and C-5 carbons of the 1,2,3-triazole ring were observed at ~140 and ~124 ppm for both compounds **1** and **2**. Unfortunately, the C-13 signals of perfluoroacyl chains were too weak to be observed, as they were split many times. ^19^F NMR (^1^H decoupling) also confirms three and eight unique types of fluorine atoms, atoms respectively in compounds **1** and **2**. Lastly, the structures of both compounds **1** and **2** confirmed by high resolution mass spectrometry (HRMS, positive electrospray). For example, compound **1** shows a peak at *m*/*z* 737.4189 which correlates well to the calculated *m*/*z* of expected proton adduct of compound **1** (C_37_H_55_F_7_N_4_O_3_, M + H^+^, *m*/*z* 737.4241). Similarly, for compound **2**, we observed a peak at *m*/*z* 987.4042 which also correlates to the calculated *m*/*z* of expected proton adduct of compound **2** (C_42_H_55_F_17_N_4_O_3_ (M + H^+^), *m*/*z* 987.4075).

### 2.2. Mesomorphic Properties

The phase behavior of compounds **1** and **2** are summarized in Table 1. Both compounds were examined by differential scanning calorimetry (DSC), polarized optical microscopy (POM), and X-ray diffraction (XRD).

**Figure 2:**
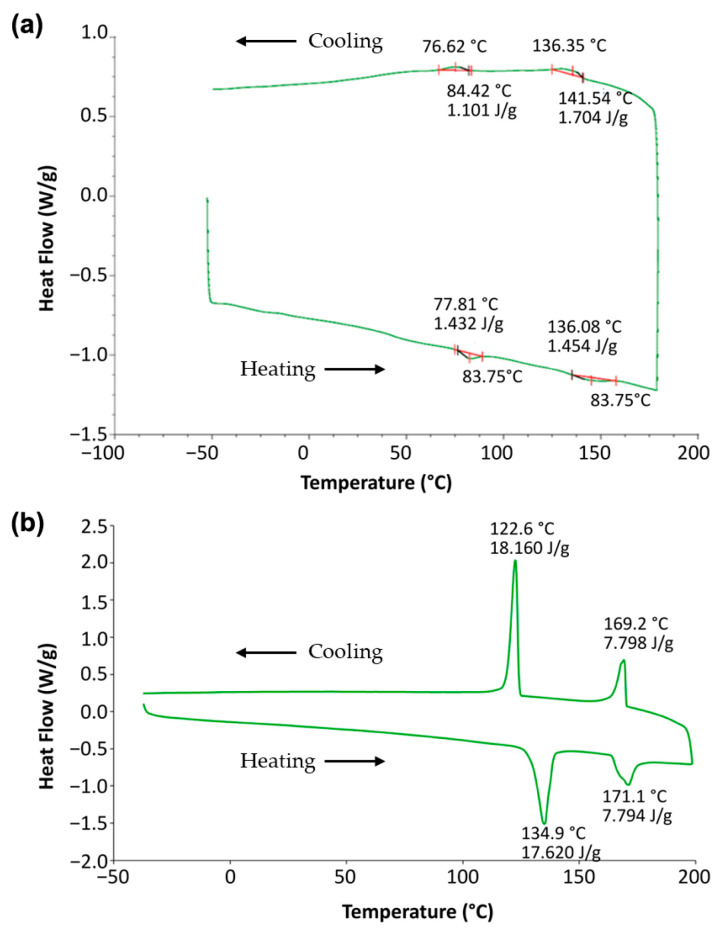
DSC thermogram of compounds **1** (**a**) and **2** (**b**) on the second heating/cooling cycle at a scan rate of 10 °C·min^−1^.

**Figure 3:**
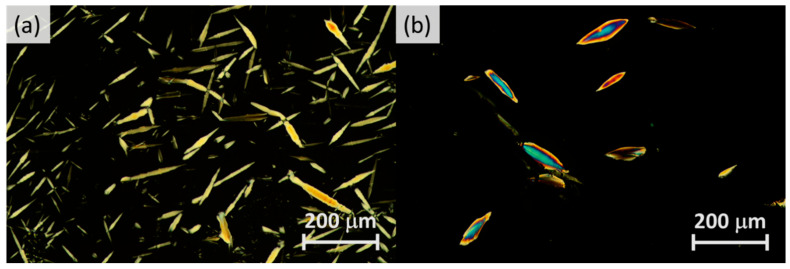
Polarized optical micrographs of **2** at (**a**) 170 °C and (**b**) 82 °C.

Compound **2** shows two reversible transitions by DSC (Figure 2b): a large enthalpy peak at 134.9 °C (13.0 kJ/mol) and a smaller peak at 171.1 °C (5.7 kJ/mol). These peaks are consistent with solid-LC (melting) and LC-isotropic (clearing) transitions, respectively. Slow cooling of the sample from the isotropic phase revealed birefringent smectic A (SmA) bâtonnet-like textures by POM (Figure 3a). These textures were highly fluid upon mechanical shearing, confirming liquid crystallinity. Upon cooling below 120 °C, the shape and birefringence of the textures changed (Figure 3b). The textures were no longer fluid and developed fissures within the film when sheared, suggesting the formation of a solid phase.

**Figure 4:**
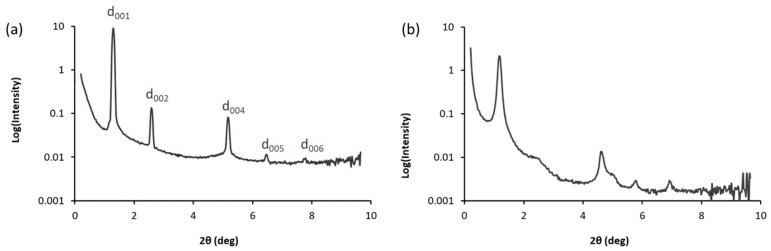
Wide-angle X-ray diffractogram of **2** at (**a**) 165 °C and (**b**) 25 °C.

**Figure 5:**
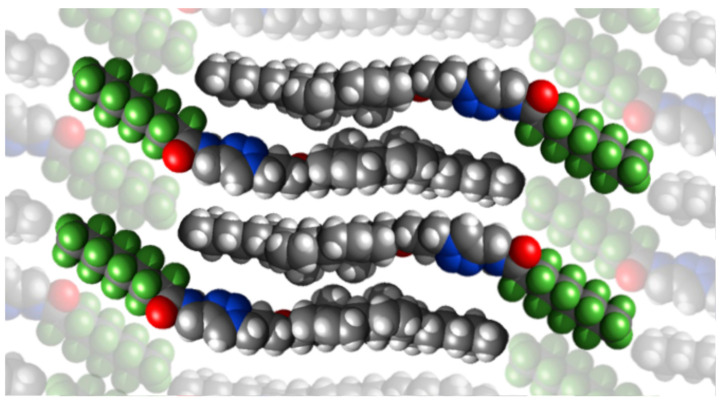
Schematic representation of the proposed ordering within the smectic phase of **2**. Carbon: dark grey; Hydrogen: light grey; Oxygen: red; Nitrogen: blue; Fluorine: green.

The X-ray diffraction pattern of **2** at 165 °C exhibits five peaks at small angles that index to the (001), (002), (004), (005) and (006) peaks of lamellar SmA* phase (Figure 4a). The layer spacing of 32.1 Å is slightly shorter than the calculated molecular length of 38 Å. The spacing suggests that the smectic phase adopts a monolayer structure in which the fluorinated side chains are interdigitated, consistent with the tendency of these chains to segregate (Figure 5). The well-defined lamellar spacing of the SmA* phase is lost as the sample cools below 130 °C, with multiple broad peaks appearing at both low and high angles (Figure 4b). This is consistent with the conversion of the SmA* phase into a crystalline solid phase at lower temperatures.

**Figure 6:**
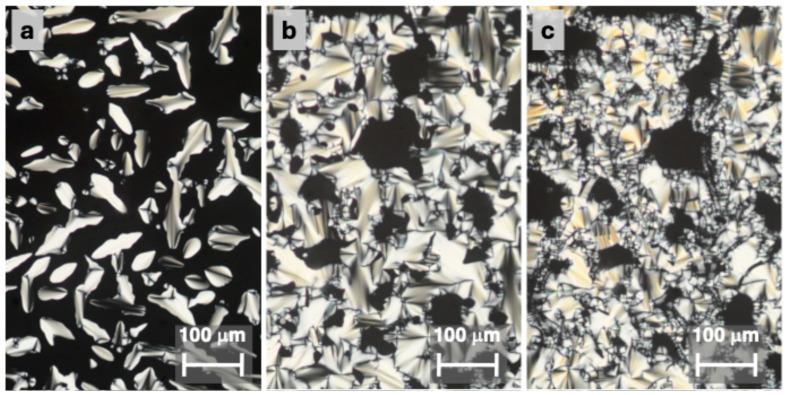
POM images of **1** on cooling from the isotropic phase at (**a**) 139 °C, (**b**) 115 °C and (**c**) 25 °C.

In broad terms, compound **1** exhibits similar phase behavior to **2**, with DSC traces that show low enthalpy reversible transitions (Figure 2a) at 83.8 °C (1.1 kJ/mol) and 145.9 °C (1.1 kJ/mol). POM observations indicate that **1** is optically isotropic above 146 °C; cooling below this temperature leads to the formation of a birefringent phase that readily flows under applied pressure, confirming that the higher temperature DSC peak is a LC-isotropic (clearing) transition. Directly below this transition, the mesophase exhibits bâtonnet domains, consistent with a SmA* phase (Figure 6a). Upon further cooling, these domains merge into a smectic fan texture (Figure 6b). This texture undergoes subtle changes between 70 and 85 °C and remains unchanged down to room temperature (Figure 6c). Notably, fluidity is lost at these lower temperatures, indicating the formation of a solid phase.

**Figure 7:**
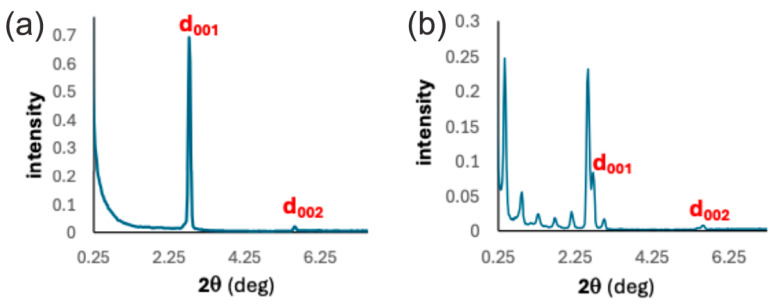
Medium angle x-ray (MAX) scattering patterns for compound **1** at 115 °C (**a**) and 60 °C (**b**). The (001) and (002) peaks of the SmA* phase are labeled in red.

Upon heating a neat sample of **1** to 115 °C, two peaks are observed in the low-angle region of the X-ray diffractogram that index to the (001) and (002) diffractions of a lamellar phase, confirming the identity of the liquid crystal as a smectic phase (Figure 7a). The interlayer spacing of 31.9 Å closely matches the calculated molecular length (32.5 Å) and is consistent with a SmA* phase with a monolayer arrangement. The layer spacing in this system is only slightly smaller than that of **2**, despite the latter possessing a substantially longer fluorinated acyl chain. It therefore appears that **1** assembles into a layered structure with substantially less interdigitation than compound **2**.

Samples cooled from the isotropic phase of **1** develop a more complex XRD pattern. At 60 °C, the (001) and (002) peaks of the SmA* phase were observed as on heating, but with the addition of eight sharp peaks at lower angles that index to the (001) to (008) peaks of a second lamellar phase. Remarkably, these new peaks correspond to an interlayer distance of 212 Å, which is approximately seven times the molecular length, suggesting a complex architecture at over a much longer scale than in the SmA* liquid crystalline phase. Notably, the XRD pattern remains largely unchanged upon further cooling to 60 °C (Figure 7b) and 25 °C, except for a slight decrease in the intensity of the SmA* peaks relative to those of the larger periodicity peaks. This observation suggests that the two distinct layer spacings arise from the coexistence of two phases: the SmA* phase and an ordered lamellar solid with a longer periodicity. We speculate that this solid phase crystallizes slowly on cooling, and that compound **1** likely exhibits a complex thermal history that depends strongly on the rates of cooling and the boundary conditions of the samples.

Comparing to the series of compounds reported by Jiang and co-workers [20] (Figure 1), the compounds reported in the current work clearly showed very different mesomorphic behavior, despite all of them being modified by perfluorinated alkyl chain. Clearly, the incorporation of a 2-hydroxy-3-(4-aminomethyl-1*H*-1,2,3-triazol-1-yl)propyl linker plays a crucial role. The incorporation of a polar hydroxyl group and an amide functionality likely provides opportunities for additional intermolecular hydrogen-bond networks to occur between adjacent monomers in the solid states. Additionally, the increased length of the linker also creates additional bond rotations, contributing to enhanced flexibility of the molecule. Both hydrogen bonding and chain flexibility are key factors known to influence liquid crystallinity.

## 3. Materials and Methods

### 3.1. Methods

**General methods.** Unless otherwise noted, commercial reagents and solvents were used without further purification. Analytical thin-layer chromatography (TLC) was carried out on silica gel 60 F254 plates (Sigma-Aldrich^®^, Oakville, ON, Canada). Visualization was achieved either by UV fluorescence quenching or by staining with a solution of aqueous sulfuric acid (5%) or a ceric ammonium molybdate, followed by heating. Flash column chromatography was performed on silica gel 60 (SiliCycle, Québec, QC, Canada). Organic extracts were concentrated under reduced pressure with the assistance of heat.

**NMR.** ^1^H NMR spectra were recorded at 400 MHz, ^13^C NMR at 100 MHz, and ^19^F NMR at approximately 376 MHz on a Bruker spectrometer (Bruker, Billerica, MA, USA). Chemical shifts (δ, ppm) are reported relative to residual solvent peaks of CDCl_3_ (δ_H_ 7.24, δ_C_ 77.0). Coupling constants (*J*) are given in Hz. gCOSY, and gHSQC experiments were used to assist the spectral assignments. High-resolution mass spectra (HRMS) were acquired on an Agilent 6520 accurate-mass QTOF LC/MS system with electrospray ionization (ESI).

**DSC**. Differential scanning calorimetry was performed on a TA Instruments Q200 calorimeter equipped with a refrigerated cooling system (RCS90) (TA Instruments, New Castle, DE, USA). Samples were heated and cooled at 10 °C/min. Due to thermal history of the samples, the data from first heating/cooling cycle were ignored.

**POM**. Polarized optical microscopy experiments was carried out on an Olympus BX50 microscope equipped with a Nikon D90 DSLR camera (Nikon, Tokyo, Japan). Temperature control was provided by a Linkam LTS350 hot stage connected to a TMS94 controller (Linkam Scientific, Redhill, UK).

**XRD**. X-Ray Diffraction measurements were obtained using a SAXSLAB Ganesha 300XL SAXS instrument (Cu source, 45 kV, 0.6 mA, Skovlunde, Denmark). Samples were sealed in thin-walled quartz capillaries (Charles Supper Company, Westborough, MA, USA, 1.5 mm o.d.) and analyzed on a Linkam T95-PE heating stage. Each diffraction pattern was collected with an exposure time of 8 min.

### 3.2. Chemical Synthesis

Compound **8**

Propargylamine (0.41 mL, 7.10 mmol) was added to methyl heptafluorobutyrate (**5**, 1.00 mL, 6.45 mmol) in methanol (5 mL) and stirred overnight. The mixture was evaporated and purified by column chromatography on silica gel (DCM: hexanes, 25:75) to obtain compound **8** as a clear oil (1.49 g, 92%). ^1^H NMR (400 MHz, CDCl_3_) *δ* 7.37 (broad, 1H, N*H*CH_2_C≡CH), 4.16 (dd, 2H, *J* = 2.5, 5.5, NHC*H*_2_C≡CH, 2.30 (m, 1H, NHCH_2_C≡C*H*). ^13^C NMR (100 MHz, CDCl_3_) *δ* 157.7 (t, *J* = 27.1 Hz, CF_2_*C*ONH), 117.3 (tq, *J* = 269.6, 33.5 Hz, *C*F_3_CF_2_), 108.4 (tt, *J* = 267.0, 31.7 Hz, CF_2_*C*F_2_CONH), 108.0 (ttq, *J* = 266.9, 39.0, 33.7 Hz, CF_3_*C*F_2_CF_2_), 76.9 (NHCH_2_C≡*C*H), 72.6 (NHCH_2_*C*≡CH), 29.7 (NH*C*H_2_C≡CH). ^19^F NMR (400 MHz, CDCl_3_) *δ* −81.1 (m, 3F, CF_3_), −121.2 (m, 2F, CF_2_), −127.4 (m, 2F, CF_2_).

Compound **9**

Propargylamine (0.50 mL, 10.6 mmol) was added to methyl perfluorononanoate (**6**, 3.27 g, 6.84 mmol) in methanol (10 mL) and stirred for 36 h before being evaporated in vacuo. The crude compound was purified by column chromatography on silica gel (DCM: hexanes, 25:75 → 35:65) to afford compound **9** as a white solid (2.53 g, 74%). R_f_ = 0.13 (DCM: hexanes, 30:70). ^1^H NMR (400 MHz, CDCl_3_) *δ* 6.68 (broad, 1H, N*H*CH_2_C≡CH), 4.21 (dd, 2H, *J* = 2.5, 5.4 Hz, NHC*H*_2_C≡CH), 2.36 (t, 1H, *J* = 2.5 Hz, NHCH_2_C≡C*H*). ^13^C NMR (100 MHz, CDCl_3_) *δ* 157.3 (t, *J* = 26.2 Hz, CF_2_*C*ONH), 120–105 (m, *C*F_3_(*C*F_2_)_7_CONH), 76.9 (NHCH_2_C≡*C*H), 73.3 (NHCH_2_*C*≡CH), 29.9 (NH*C*H_2_C≡CH). ^19^F NMR (400 MHz, CDCl_3_) *δ* −80.8 (t, *J* = 10.1 Hz, 3F, CF_3_), −119.7 (t, 2F, *J* = 12.7 Hz, CF_2_), −121.5 (m, 2F, CF_2_), −121.9 (m, 4F, 2 × CF_2_), −122.5 (m, 2F, CF_2_), −122.7 (m, 2F, CF_2_), −126.1 (m, 2F, CF_2_).

Compound **4**

Compound **4** was prepared according to the cited literature [24].

Compound **5**

Compound **4** (50.6 mg, 0.11 mmol) was added to a solution of sodium azide (45 mg, 0.69 mmol) dissolved in dimethylformamide (1.00 mL). The reaction was stirred at 80 °C for 48 h before being diluted with ethyl acetate (~50 mL). The organic layer was washed with water (50 mL × 2), evaporated and co-evaporated with toluene under reduced pressure. The crude mixture was purified by column chromatography on silica gel (ethyl acetate:hexanes, 4:96) to obtain compound **5** as a white solid (31.07 mg, 56%). R_f_ = 0.34 (EtOAc:hexanes, 20:80). Selected ^1^H NMR (400 MHz, CDCl_3_) *δ* 5.37 (m, 1H, HC=C), 3.92 (m, 1H, HOC*H*), 3.57 (m, 1H, OC*H*_a_H*_b_*), 3.50 (m, 1H, OCH_a_*H*_b_), 3.46–3.33 (m, 2H, OCH_2_N_3_), 3.22 (m, 1H, OCH), 2.48 (br s, 1H, OH), 2.38 (m, 1H, OCHC*H*_a_H*_b_*C=CH), 2.22 (m, 1H, OCHCH_a_*H_b_*C=CH). ^13^C NMR (101 MHz, CDCl_3_) *δ* 140.5 (HC=*C*), 122.0 (H*C*=C), 79.9 (OCH), 69.8 (HOCH), 69.0 (OCH_2_), 56.8, 56.2, 53.5 (CH_2_N_3_), 50.2, 42.3, 39.8, 39.5, 39.1 (OCH*C*H_2_C=C), 39.0, 37.1, 36.9, 36.2, 35.8, 32.0, 31.9, 29.7, 28.4, 28.3, 28.2, 28.0, 24.3, 23.8, 22.8, 22.6, 21.1, 19.4, 18.7, 11.9. HRMS (ESI-QTOF, positive) *m*/*z* calc’d for C_30_H_51_N_3_O_2_ (M + Na^+^): 508.3873; found: 508.3851 (Δ = 4.3 ppm).

Compound **1**

Compound **5** (9.0 mg, 18.5 µmol) was dissolved in acetone (2.0 mL). Compound **8** (9.0 mg, 37 µmol) was added along with DIPEA (0.3 µL, 1.9 µmol) and CuI (0.4 mg, 1.9 µmol) under an inert argon atmosphere. The reaction was stirred for 12 h before being evaporated under reduced pressure. The crude mixture was dissolved in ethyl acetate (50 mL) and washed with 5% *N*,*N*,*N′*,*N′-*ethylenediaminetetraacetic acid in water (50 mL × 2). The organic phase was evaporated and co-evaporated with toluene to obtain the crude product which was purified by column chromatography on silica gel (ethyl acetate:hexanes, 30:70) to obtain the target compound **1** as a white solid (11.6 mg, 85%). R_f_ = 0.35 (EtOAc:hexanes, 60:40). Selected ^1^H NMR (400 MHz, CDCl_3_) *δ* 7.74 (s, 1H, 1,2,3-triazole), 7.28 (br s, 1H, NH), 5.37 (m, 1H, HC=C), 4.67 (d, *J* = 5.5 Hz, 2H, NHC*H*_2_), 4.56 (m, 1H, NC*H*_a_H*_b_*), 4.43 (m, 1H, NCH_a_*H_b_*), 4.17 (m, 1H, HOC*H*), 3.57 (m, 1H, OC*H*_a_H*_b_*), 3.39 (m, 1H, OCH_a_*H_b_*), 3.20 (m, 1H, OCH), 2.80 (br s, 1H, OH), 2.34 (m, 1H, OCHC*H*_a_H*_b_*C=CH), 2.21 (m, 1H, OCHCH_a_*H_b_*C=CH). ^13^C NMR (101 MHz, CDCl_3_) *δ* 140.3 (C4_1,2,3-triazole), 123.9 (C5_1,2,3-triazole), 122.1 (C=*C*H_cholesterol), 79.9 (OCH), 69.3 (HOCH), 68.6 (OCH_2_), 56.8, 56.2, 53.1 (NCH_2_), 50.2, 42.3, 39.8, 39.5, 39.0 (OCH*C*H_2_C=C), 37.0, 36.8, 36.2, 35.8 (NHCH_2_), 35.5, 31.9, 31.88, 29.7, 28.3, 28.29, 28.23, 28.0, 24.3, 23.8, 22.8, 22.6, 21.1, 19.4, 18.7, 11.9. ^19^F NMR (376 MHz, CDCl_3_) *δ* −80.6, −120.7, −126.9. HRMS (ESI-QTOF, positive) *m*/*z* calc’d for C_37_H_55_F_7_N_4_O_3_ (M + H^+^): 737.4235; found: 737.4189 (Δ = 6.2 ppm).

Compound **2**

Compound **5** (0.13 g, 2.68 mmol) was dissolved in acetone (7.0 mL). Compound **9** (0.27 g, 5.36 mmol) was added along with DIPEA (4.7 µL, 26.8 µmol) and CuI (5.1 mg, 26.8 µmol) under an inert argon atmosphere. The reaction was stirred for 12 h before being evaporated under reduced pressure. The crude mixture was dissolved in ethyl acetate (50 mL) and washed with 5% *N,N,N′,N′-*ethylenediaminetetraacetic acid in water (50 mL × 2). The organic phase was evaporated and co-evaporated with toluene to obtain the crude product which was purified by column chromatography on silica gel (ethyl acetate:hexanes, 30:70) to obtain the target compound **2** as a white solid (0.24 g, 88%). R_f_ = 0.32 (EtOAc:hexanes, 50:50). Selected ^1^H NMR (400 MHz, CDCl_3_) *δ* 7.73 (s, 1H, 1,2,3-triazole), 7.19 (br s, 1H, NH), 5.36 (m, 1H, HC=C), 4.68 (d, *J* = 5.5 Hz, 2H, NHC*H*_2_), 4.56 (m, 1H, NC*H*_a_H*_b_*), 4.43 (m, 1H, NCH_a_*H_b_*), 4.17 (m, 1H, HOC*H*), 3.57 (m, 1H, OC*H*_a_H*_b_*), 3.39 (m, 1H, OCH_a_*H_b_*), 3.20 (m, 1H, OCH), 2.78 (br s, 1H, OH), 2.35 (m, 1H, OCHC*H*_a_H*_b_*C=CH), 2.21 (m, 1H, OCHCH_a_*H_b_*C=CH). ^13^C NMR (101 MHz, CDCl_3_) *δ* 140.3 (C4_1,2,3-triazole), 123.8 (C5_1,2,3-triazole), 122.1 (C=*C*H_cholesterol), 79.9 (OCH), 69.3 (HOCH), 68.6 (OCH_2_), 56.8, 56.2, 53.0 (NCH_2_), 50.2, 42.3, 39.8, 39.5, 39.0 (OCH*C*H_2_C=C), 38.9, 37.0, 36.8, 36.2, 35.8 (NHCH_2_), 35.5, 29.7, 28.3, 28.28, 24.3, 23.8, 22.8, 22.6, 21.1, 19.4, 18.7, 11.9. ^19^F NMR (376 MHz, CDCl_3_) *δ* −80.7, −119.7, −121.5, −121.9, −122.4, −122.7, −126.1. HRMS (ESI-QTOF, positive) *m*/*z* calc’d for C_37_H_55_F_7_N_4_O_3_ (M + H^+^): 987.4075; found: 987.4042 (Δ = 3.4 ppm).

## 4. Conclusions

In this study, two novel cholesterol-based molecules bearing fluorinated chains were successfully synthesized, and their liquid crystalline properties were systematically investigated. Both compounds form smectic A phases, likely resulting from a combined effect of perfluorinated acyl chains and the 2-hydroxy-3-(4-aminomethyl-1*H*-1,2,3-triazol-1-yl)propyl linker, which both enables additional intermolecular interactions to occur. Compound **1** also forms a solid lamellar phase that, remarkably, exhibits a layer spacing of more than 200 Å, a distance that is approximately seven times the molecular length. Such a large spacing suggests a complex ordering between individual layers, which may arise due to the interplay of the fluorinated chains and the chiral structure of the building blocks. Ongoing work is focused on elucidating the detailed layer organization through different XRD and modeling studies. The combination of cholesterol and fluorinated chains provides a versatile platform to design functional soft materials that could find applications in different fields such as opto-electronics, color information technology and biotechnology.

## Figures and Tables

**Table 1:** Phase behavior of compounds **1** and **2**.

Compound	Phase ^a^	T (°C) [ΔH (kJ/mol)] ^b^	Phase ^a^	T (°C) [ΔH (kJ/mol)] ^b^	Phase ^a^
**1**	Cr	83.8 [1.1] 76.6 [−0.8]	SmA*	145.9 [1.1] 146.4 [−1.3]	Iso
**2**	Cr	134.9 [13.0] 122.6 [−13.4]	SmA*	171.1 [5.7] 169.2 [−5.7]	Iso

a. Phases identified by POM and XRD experiments. b. Transition temperatures and enthalpies were determined by DSC (scan rate = 10 °C/min) on the second heating/cooling cycle. Cr = crystalline solid, SmA* = smectic A*, Iso = isotropic. b Heating data is shown in red and cooling data is shown in blue.

## Data Availability

The data is contained within the article.

## Supplementary Materials

The following supporting information can be downloaded at: https://www.mdpi.com/article/10.3390/molecules30183731/s1. Figure S1. ^1^H NMR spectrum of compound **8**; Figure S2. ^13^C NMR spectrum of compound **8**; Figure S3. ^19^F NMR spectrum of compound **8**; Figure S4. ^1^H NMR spectrum of compound **9**; Figure S5. ^13^C NMR spectrum of compound **9**; Figure S6. ^19^F NMR spectrum of compound **9**; Figure S7. ^1^H NMR spectrum of compound **5**; Figure S8. ^13^C NMR spectrum of compound **5**; Figure S9. ^1^H-^1^H COSY NMR spectrum of compound **5**; Figure S10. ^1^H-^13^C HSQC NMR spectrum of compound **5**; Figure S11. ^1^H NMR spectrum of compound **1**; Figure S12. ^13^C NMR spectrum of compound **1**; Figure S13. ^1^H-^1^H COSY NMR spectrum of compound **1**; Figure S14. ^1^H-^13^C HSQC NMR spectrum of compound **1**; Figure S15. ^19^F NMR spectrum of compound **1**; Figure S16. ^1^H NMR spectrum of compound **2**; Figure S17. ^13^C NMR spectrum of compound **2**; Figure S18. ^1^H-^1^H COSY NMR spectrum of compound **2**; Figure S19. ^1^H-^13^C HSQC NMR spectrum of compound **2**; Figure S20. ^19^F NMR spectrum of compound **2**.
