# Supplementary material for: Synthesis and Characterization of Cholesterol-Based Liquid Crystals Linked with Perfluorinated Alkyl Chains
Source: Molecules. 2025 Sep 13;30(18):3731. doi: 10.3390/molecules30183731 (PMC12472246; doi:10.3390/molecules30183731)
Supplement: Supplementary file 1 [file molecules-30-03731-s001.zip › molecules-3832770-supplementary.pdf]

Supporting Information

Synthesis and Characterization of Cholesterol-Based Liquid Crystals Linked with Perfluorinated Alkyl Chains

Austin Che,[1] Carson O. Zellmann-Parrotta,[2] Homayoun Ghaseminezhad,[2] Jessica Duong,[1] Vance E. Williams,[2,] and Chang-Chun Ling[1,*]*

[1]Department of Chemistry, University of Calgary, Calgary, Alberta T2N 1N4, Canada

[2]Department of Chemistry, Simon Fraser University, Burnaby, British Columbia V5A 1S6, Canada

Corresponding author: ccling@ucalgary.ca (C.-C.L.); vancew@sfu.ca (V.E.W.); Fax: +1-403-289-9488 (C.-C.L.); +1-604-291-3765 (V.E.W.).

Contents

NMR Spectra 4

NMR spectra of compound **8** 4

Figure S1. ^{1}H NMR spectrum of compound **8** 4

Figure S2. ^{13}C NMR spectrum of compound **8** 5

Figure S3. ^{19}F NMR spectrum of compound **8** 6

NMR spectra of compound **9** 7

Figure S4. ^{1}H NMR spectrum of compound **9** 7

Figure S5. ^{13}C NMR spectrum of compound **9** 8

Figure S6. ^{19}F NMR spectrum of compound **9** 9

NMR spectra of compound **5** 10

Figure S7. ^{1}H NMR spectrum of compound **5** 10

Figure S8. ^{13}C NMR spectrum of compound **5** 11

Figure S9. ^{1}H-^{1}H COSY NMR spectrum of compound **5** 12

Figure S10. ^{1}H-^{13}C HSQC NMR spectrum of compound **5** 13

NMR spectra of compound **1** 14

Figure S11. ^{1}H NMR spectrum of compound **1** 14

Figure S12. ^{13}C NMR spectrum of compound **1** 15

Figure S13. ^{1}H-^{1}H COSY NMR spectrum of compound **1** 16

Figure S14. ^{1}H-^{13}C HSQC NMR spectrum of compound **1** 17

Figure S15. ^{19}F NMR spectrum of compound **1** 18

NMR spectra of compound **2** 19

Figure S16. ^{1}H NMR spectrum of **2** 19

Figure S17. ^{13}C NMR spectrum of **2** 20

Figure S18. ^1H-^1H COSY NMR spectrum of compound **2** 21
Figure S19. ^1H-^{13}C HSQC NMR spectrum of compound **2** 22
Figure S20. ^{19}F NMR spectrum of compound **2** 23

NMR Spectra

*NMR spectra of compound **8***

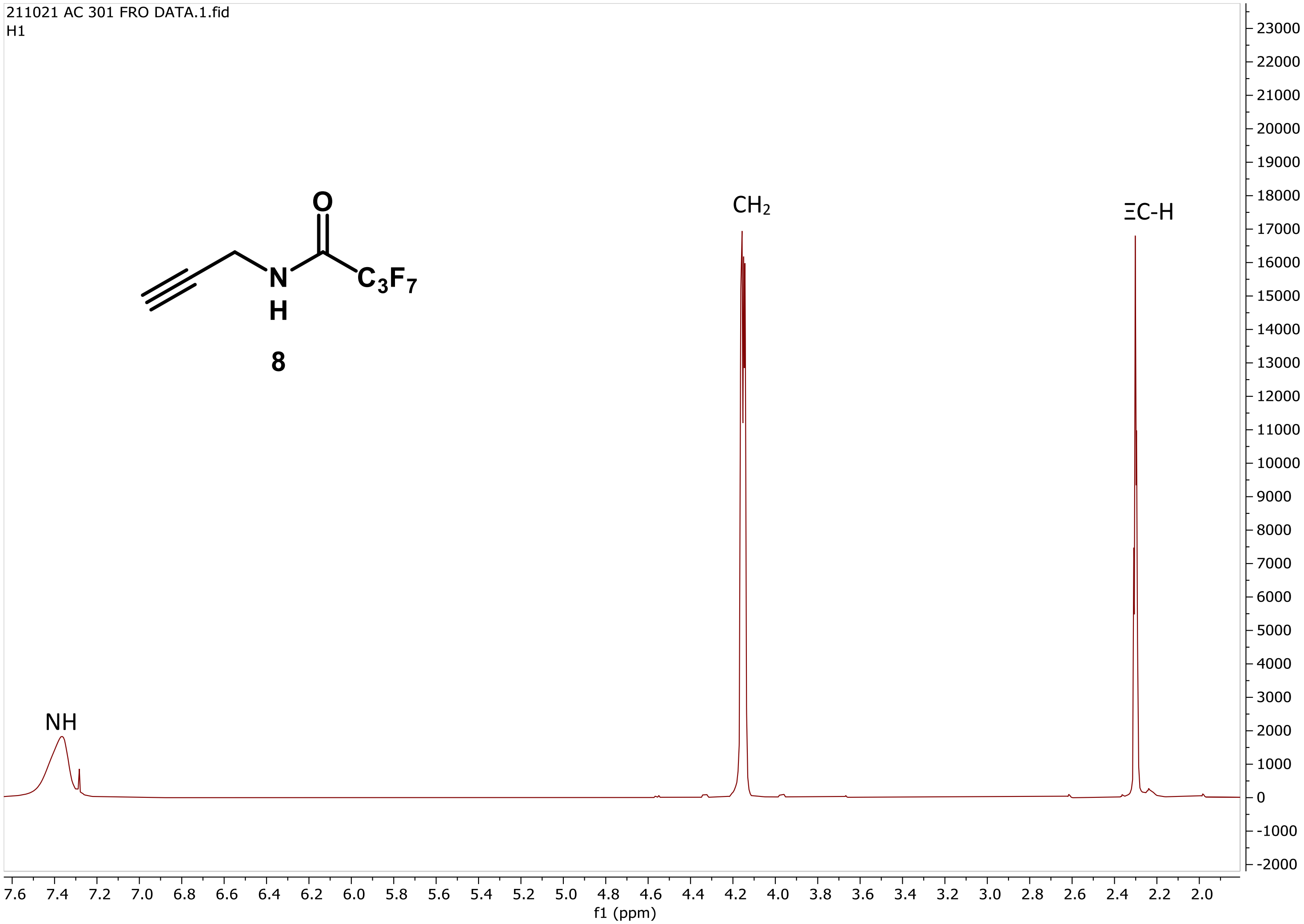

Figure S1. ^{1}H NMR spectrum of compound **8**.

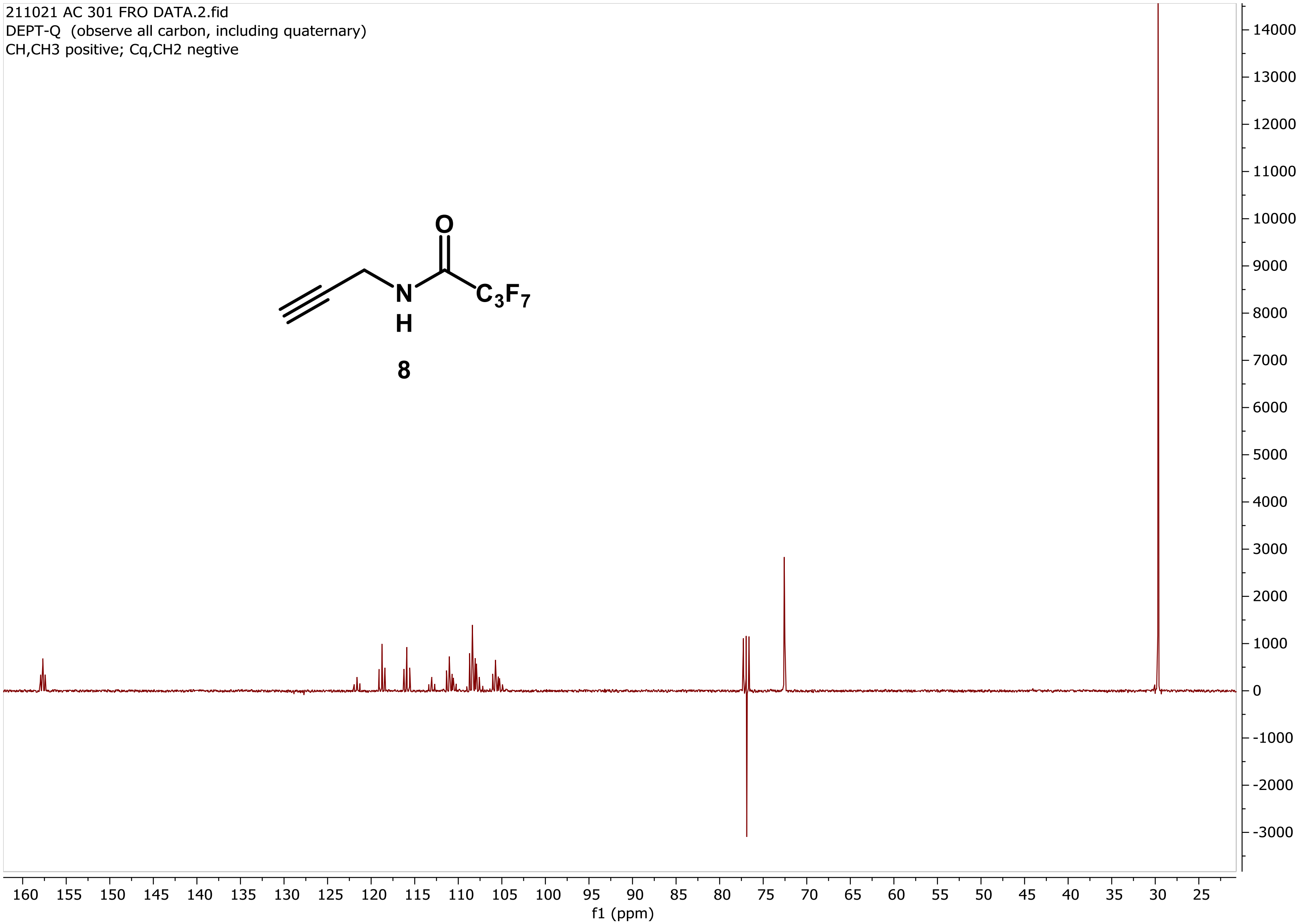

Figure S2. ^{13}C NMR spectrum of compound **8**.

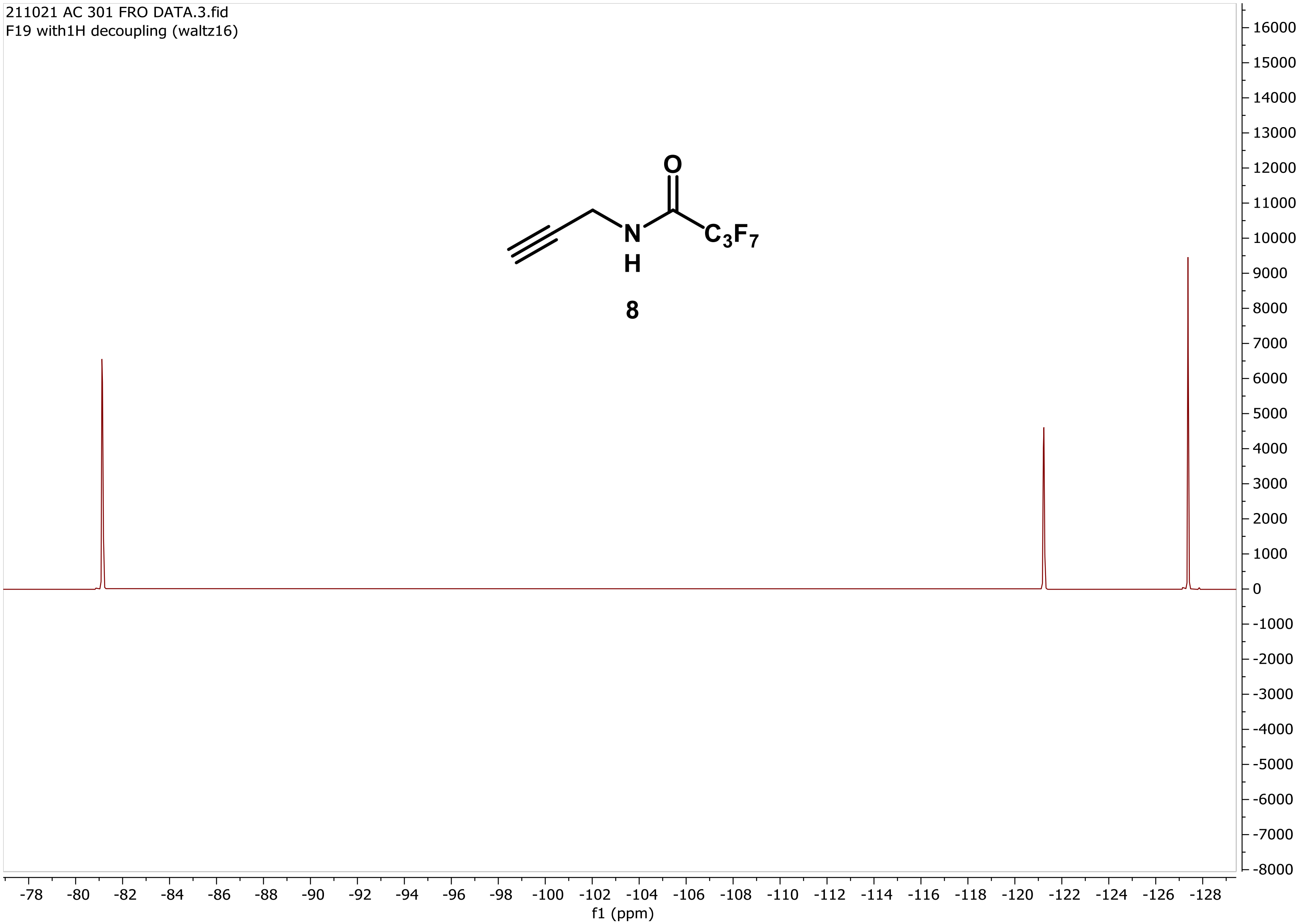

Figure S3. ^{19}F NMR spectrum of compound **8**.

*NMR spectra of compound **9***

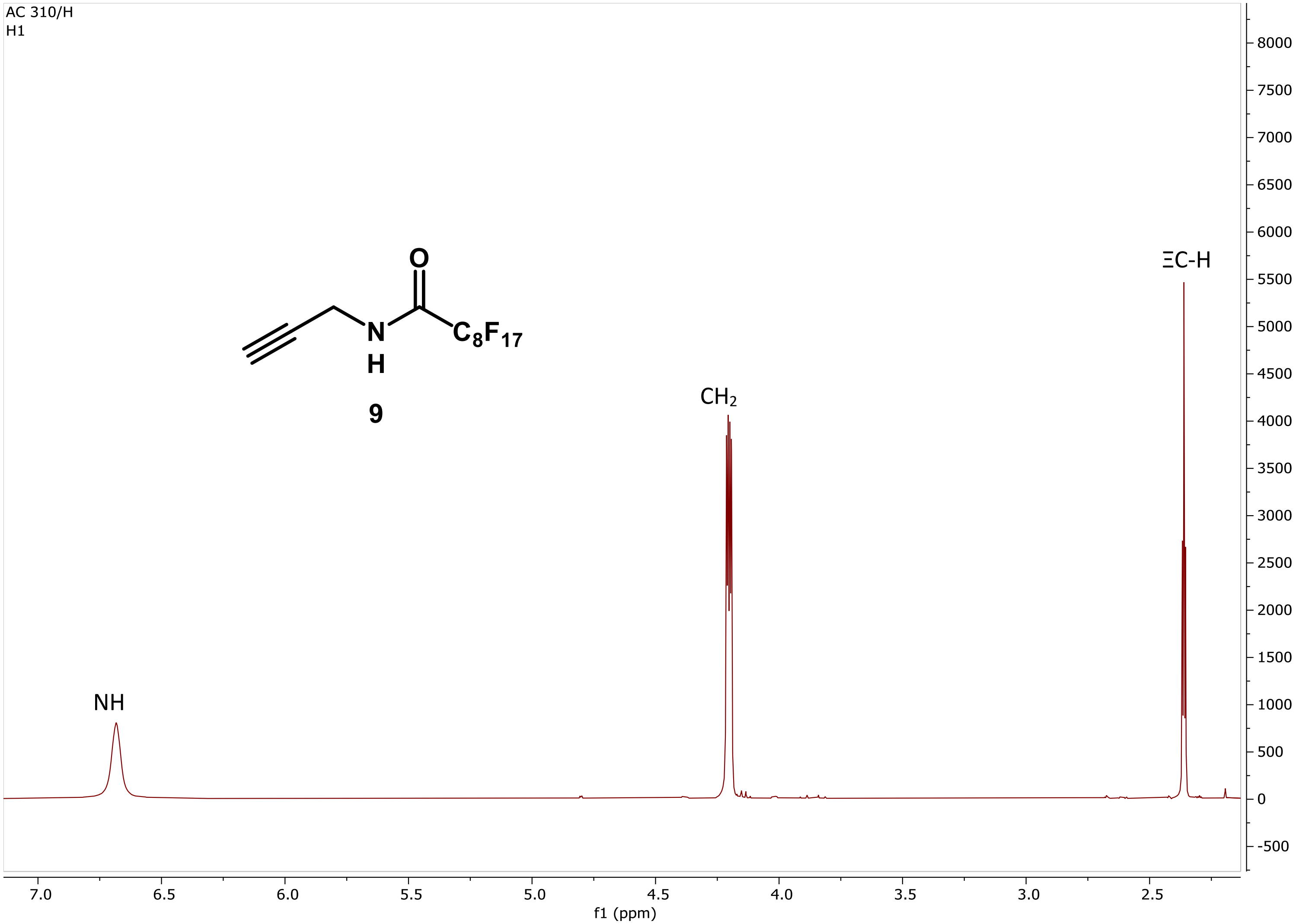

Figure S4. ^{1}H NMR spectrum of compound **9**.

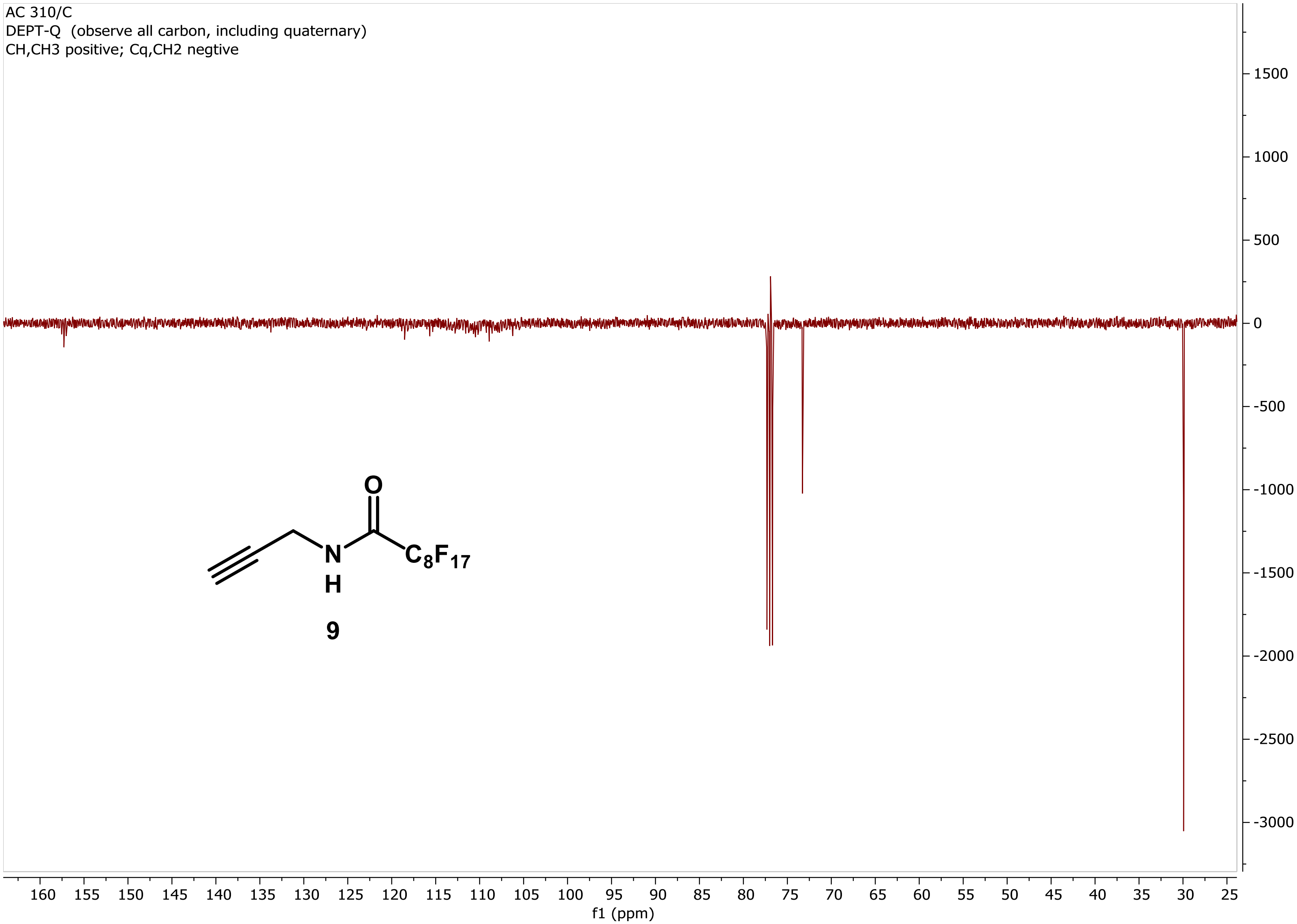

Figure S5. ^{13}C NMR spectrum of compound **9**.

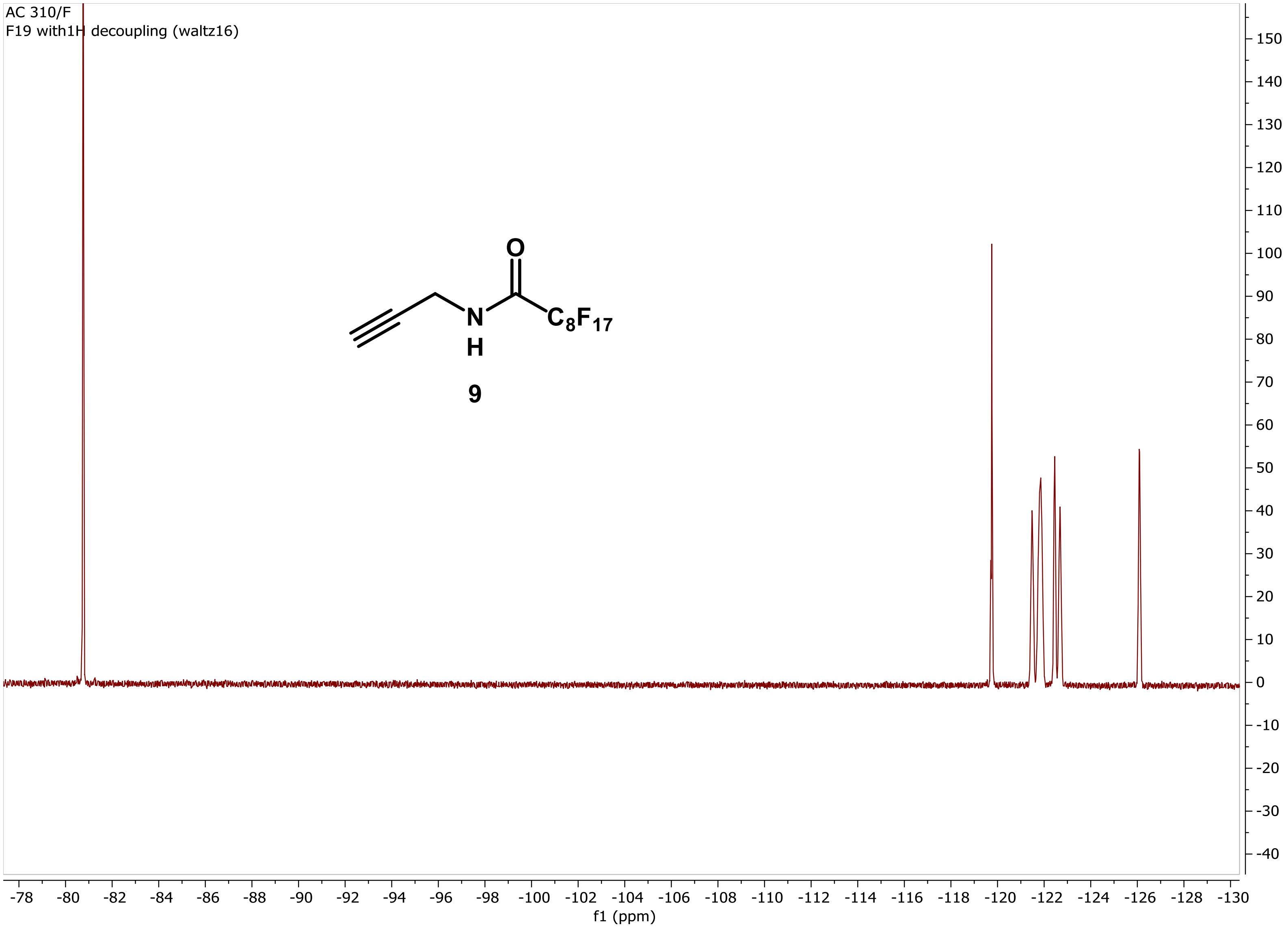

Figure S6. ^{19}F NMR spectrum of compound **9**.

NMR spectra of compound ***5***

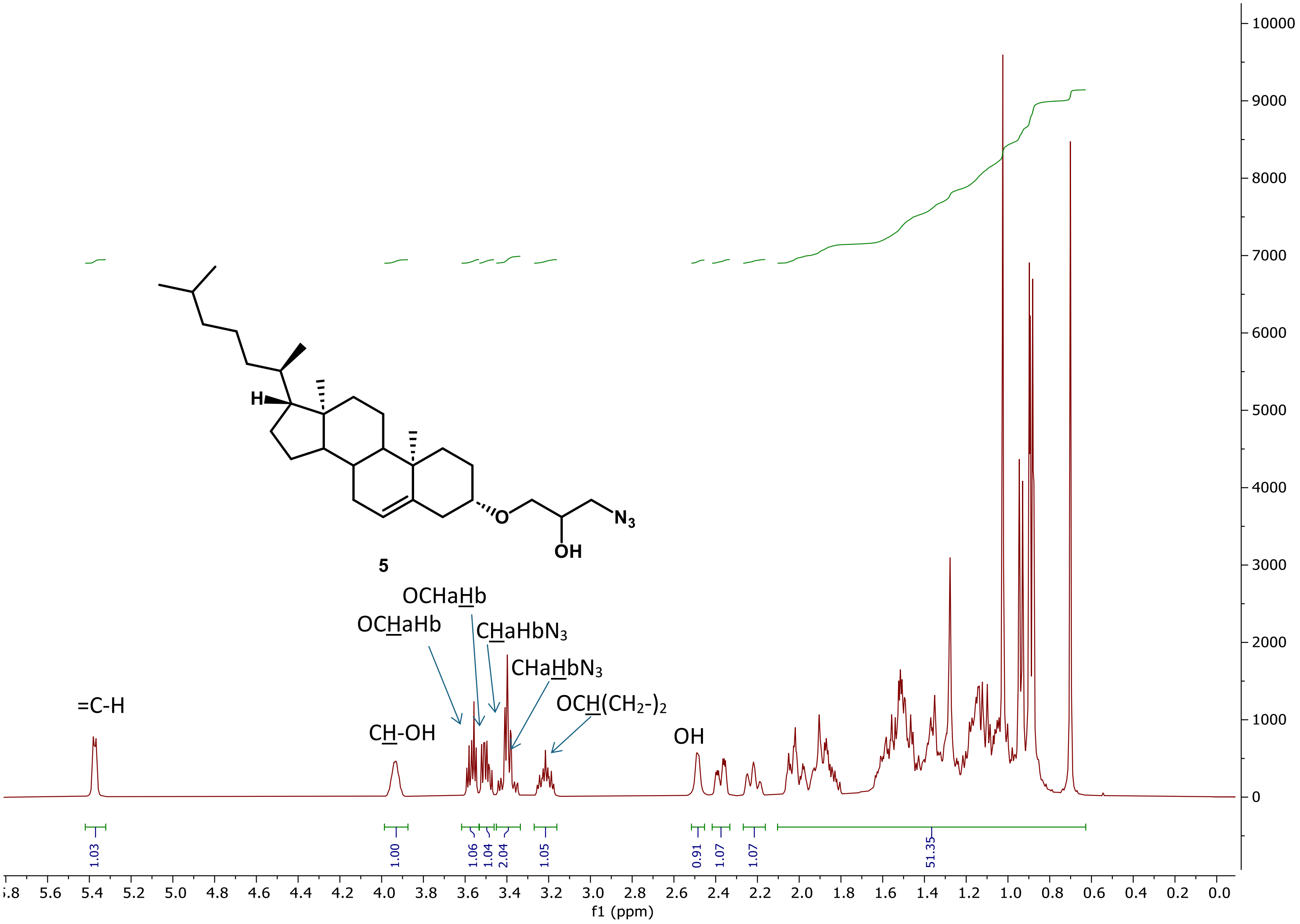

Figure S7. ^{1}H NMR spectrum of compound **5**.

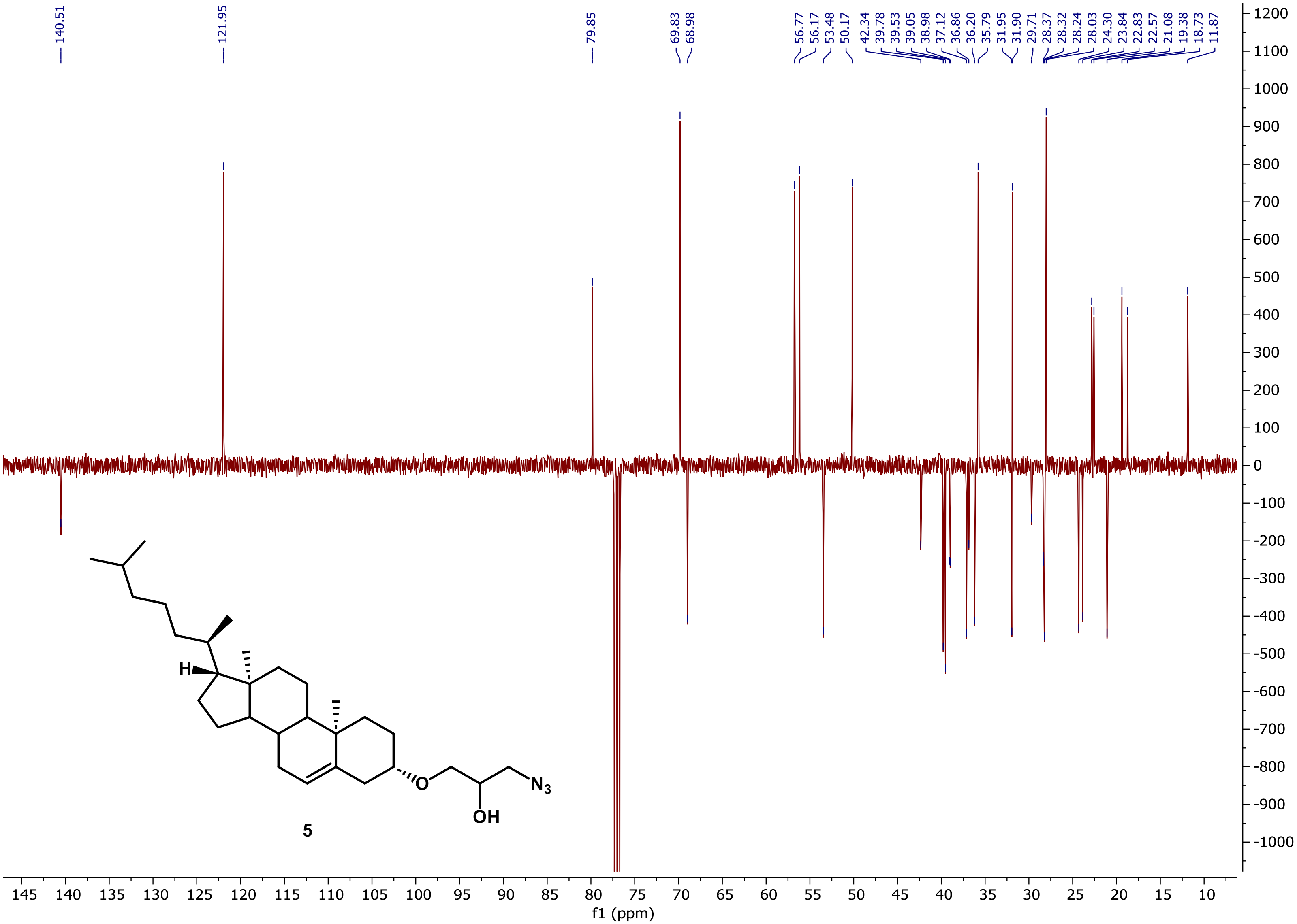

Figure S8. ^{13}C NMR spectrum of compound **5**.

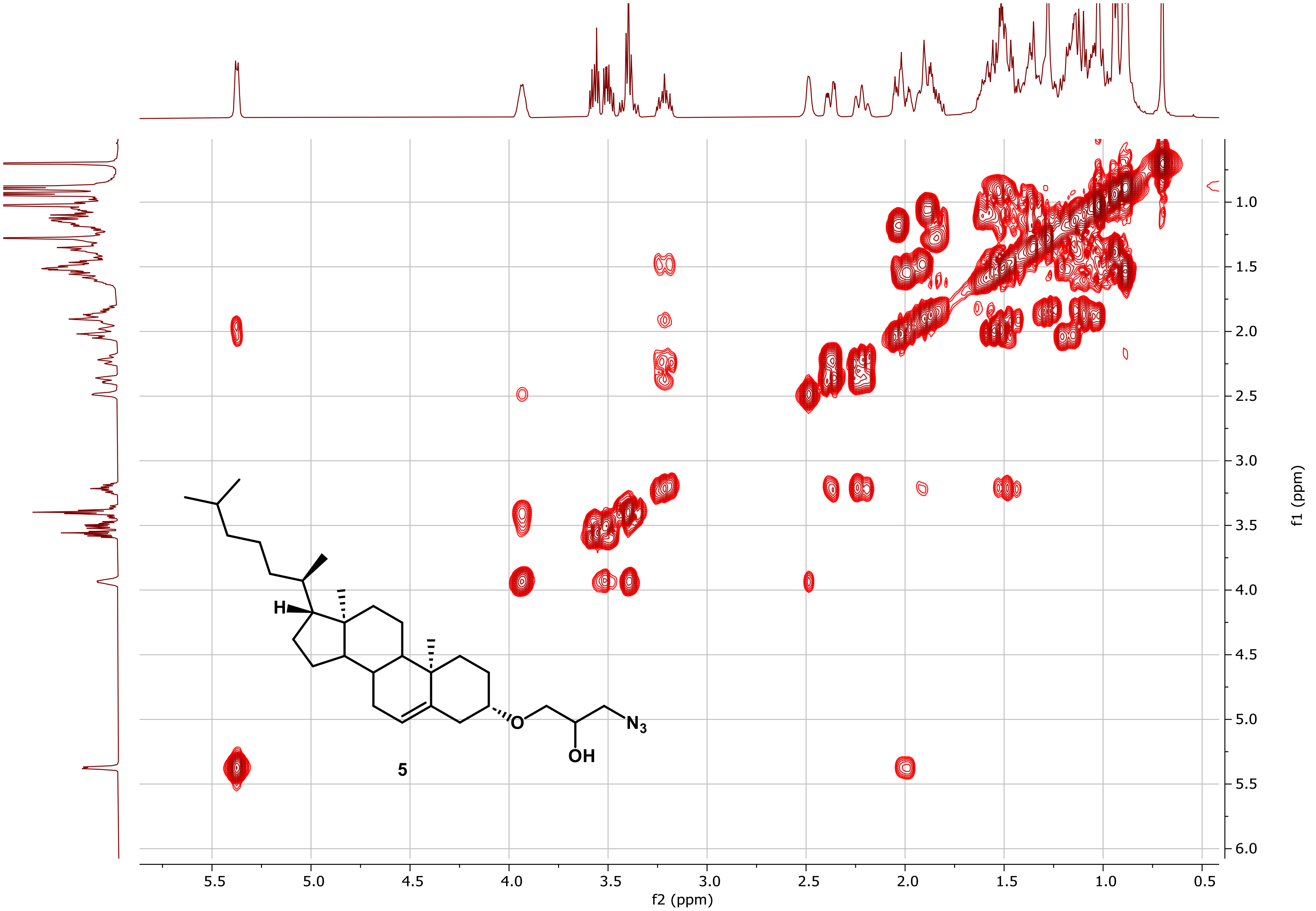

Figure S9. ^{1}H-^{1}H COSY NMR spectrum of compound **5**.

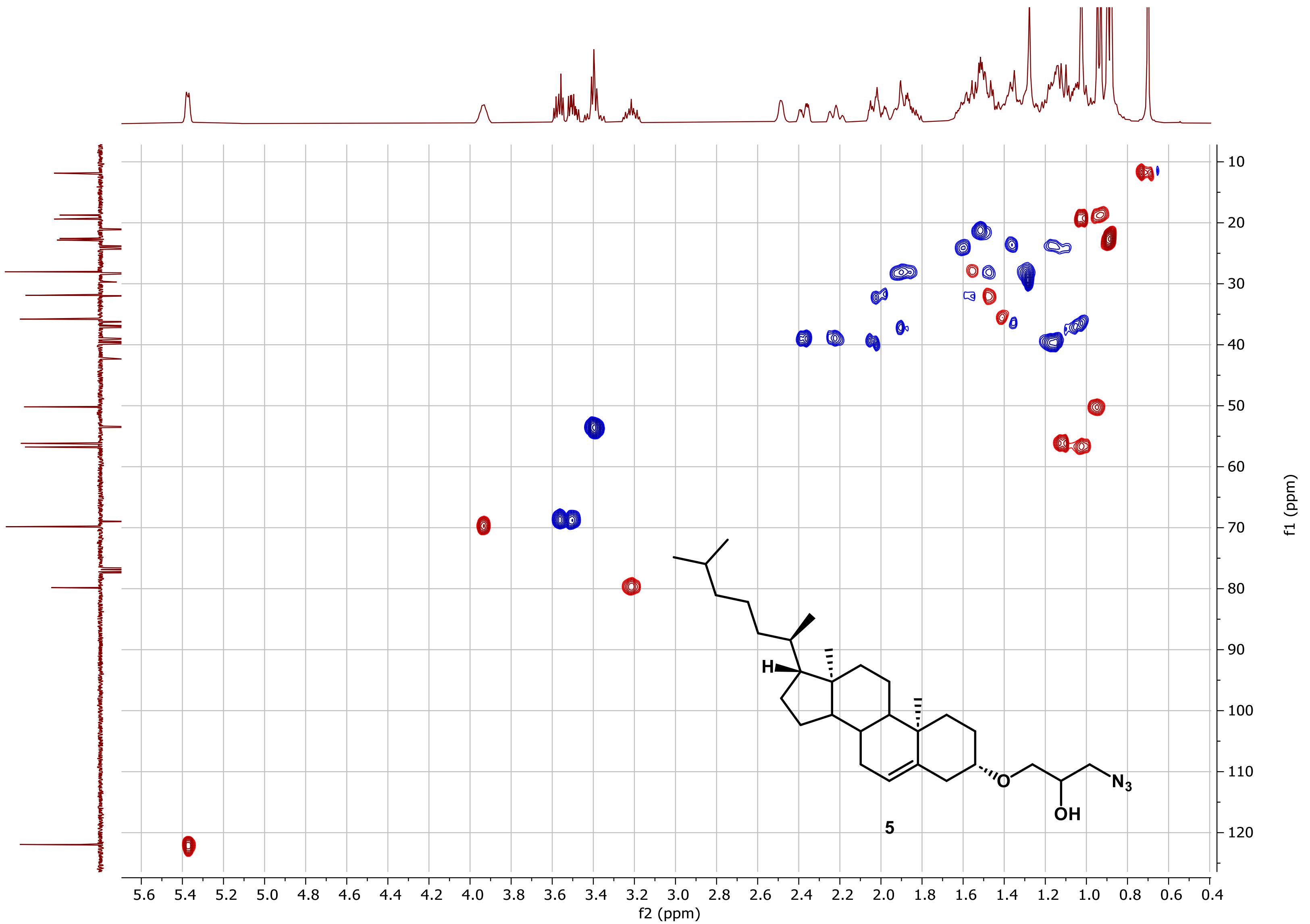

Figure S10. ^{1}H-^{13}C HSQC NMR spectrum of compound **5**.

*NMR spectra of compound **1***

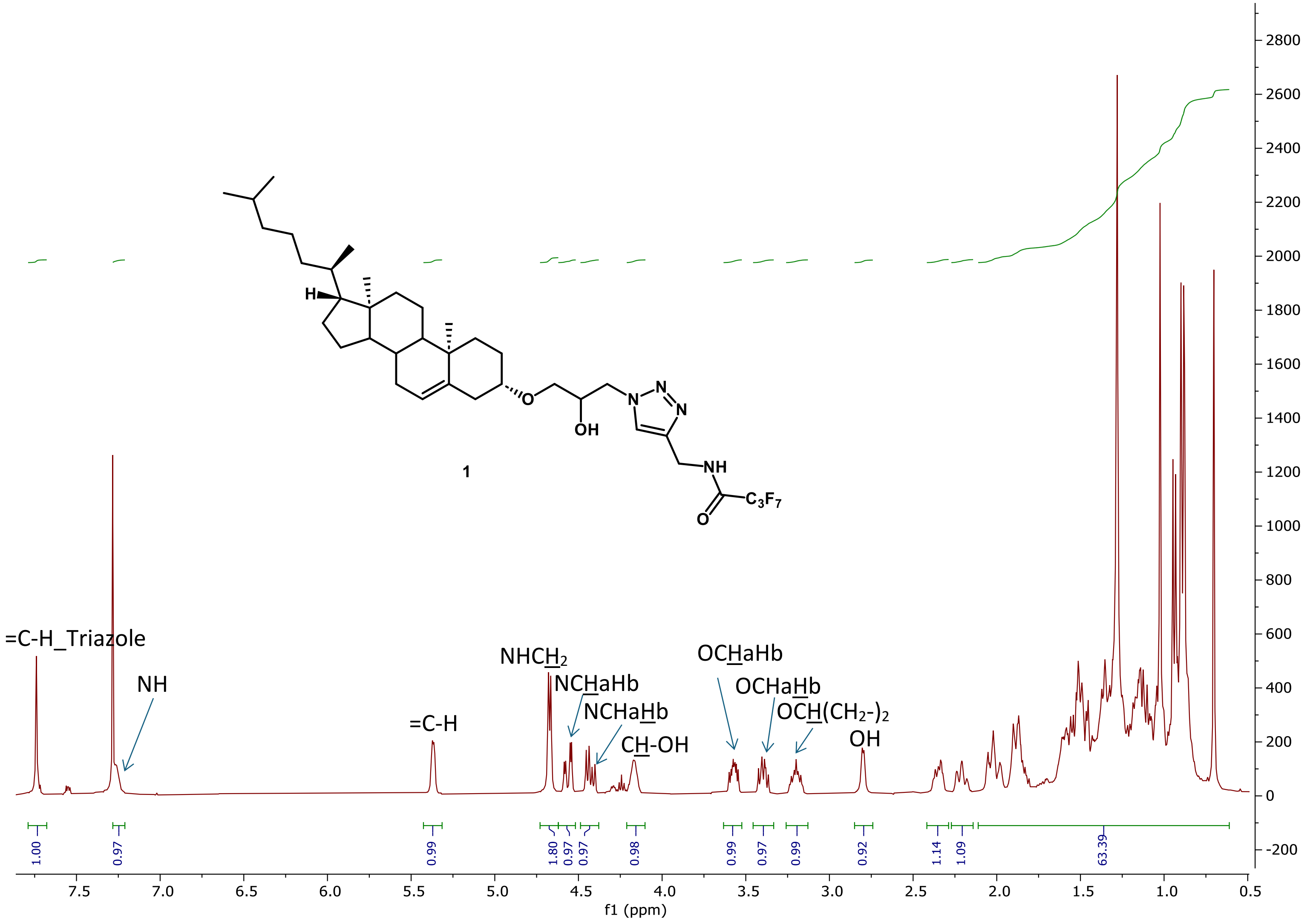

Figure S11. ^{1}H NMR spectrum of compound **1**.

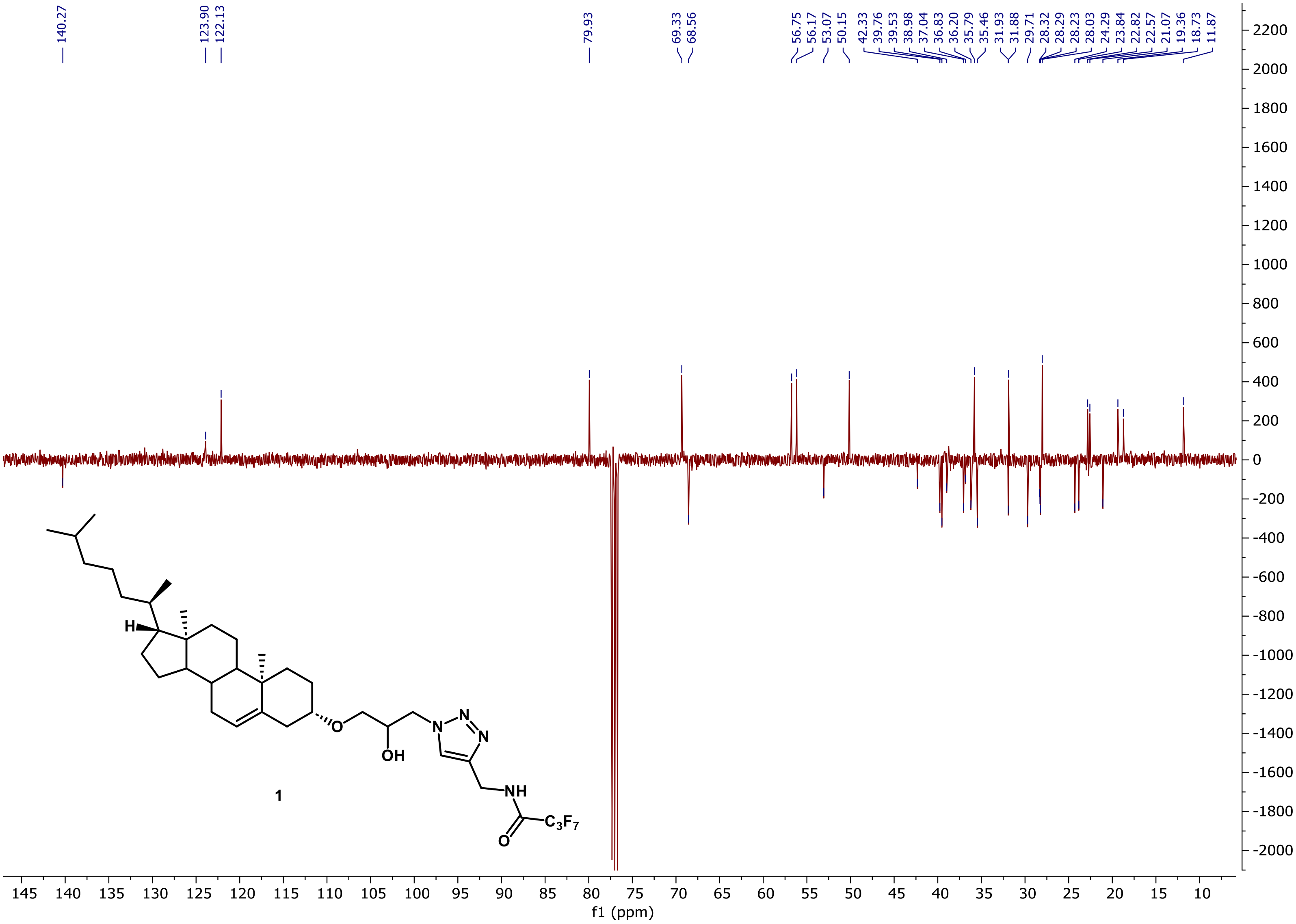

Figure S12. ^{13}C NMR spectrum of compound **1**.

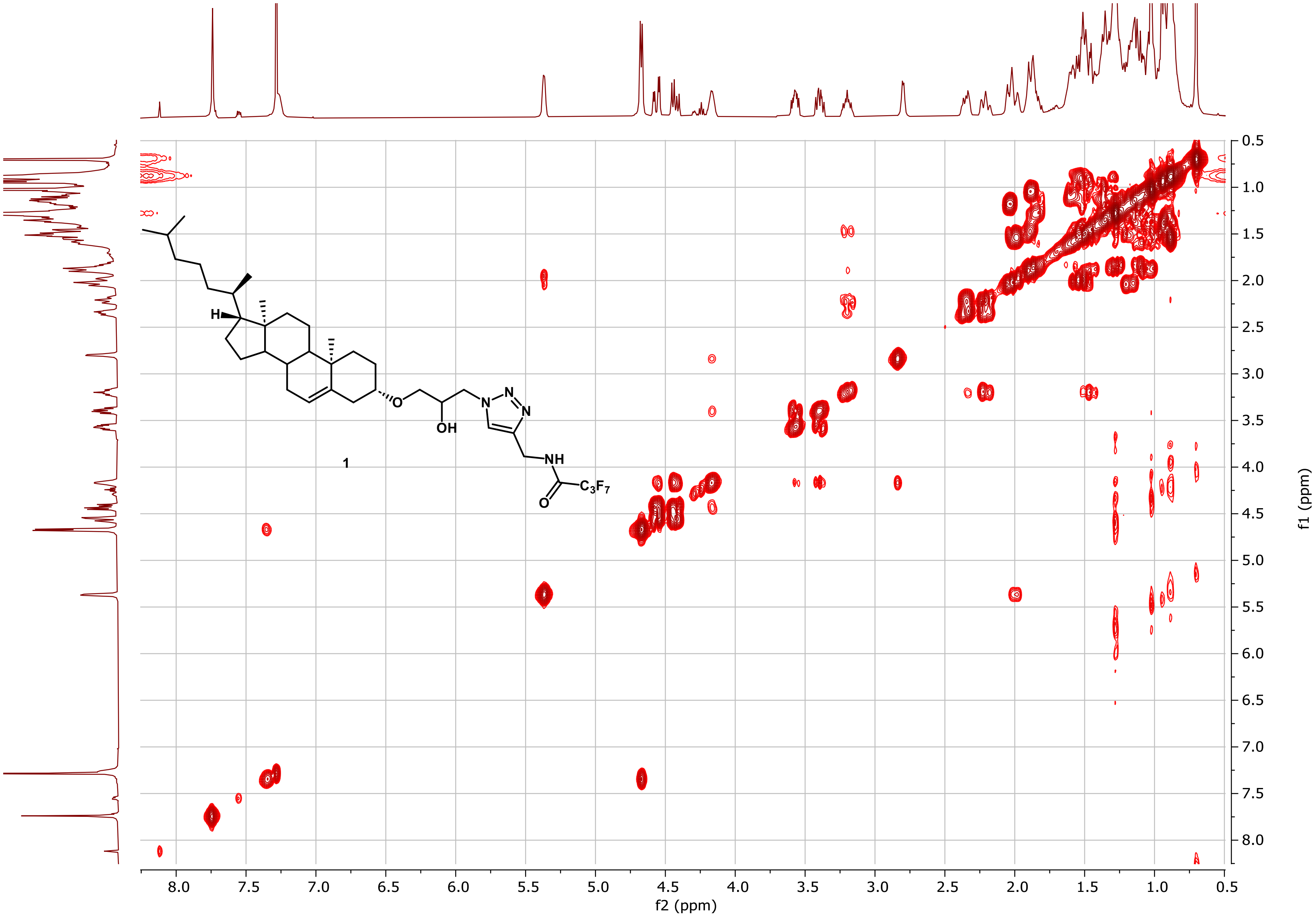

Figure S13. ^{1}H-^{1}H COSY NMR spectrum of compound **1**.

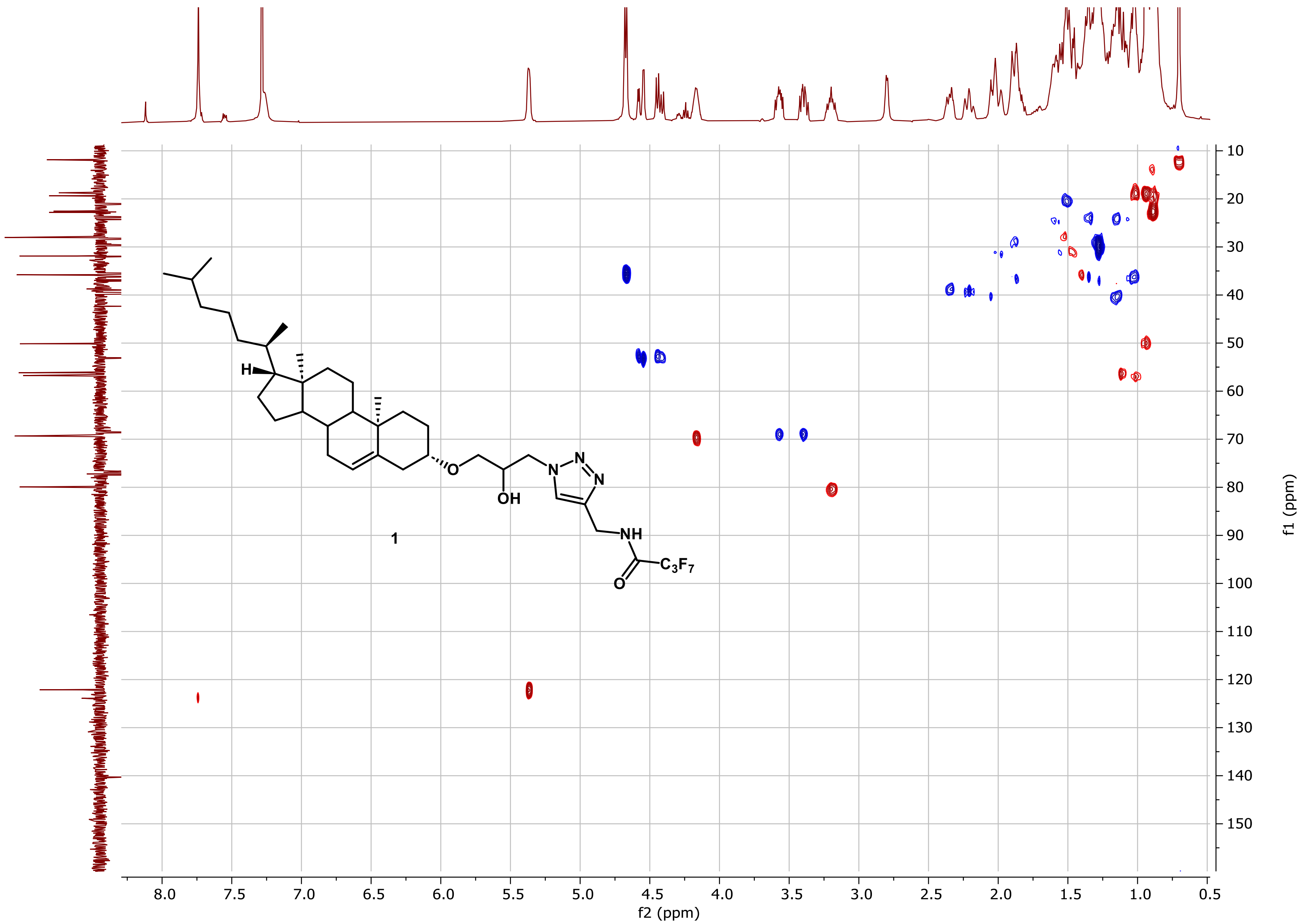

Figure S14. ^{1}H-^{13}C HSQC NMR spectrum of compound **1**.

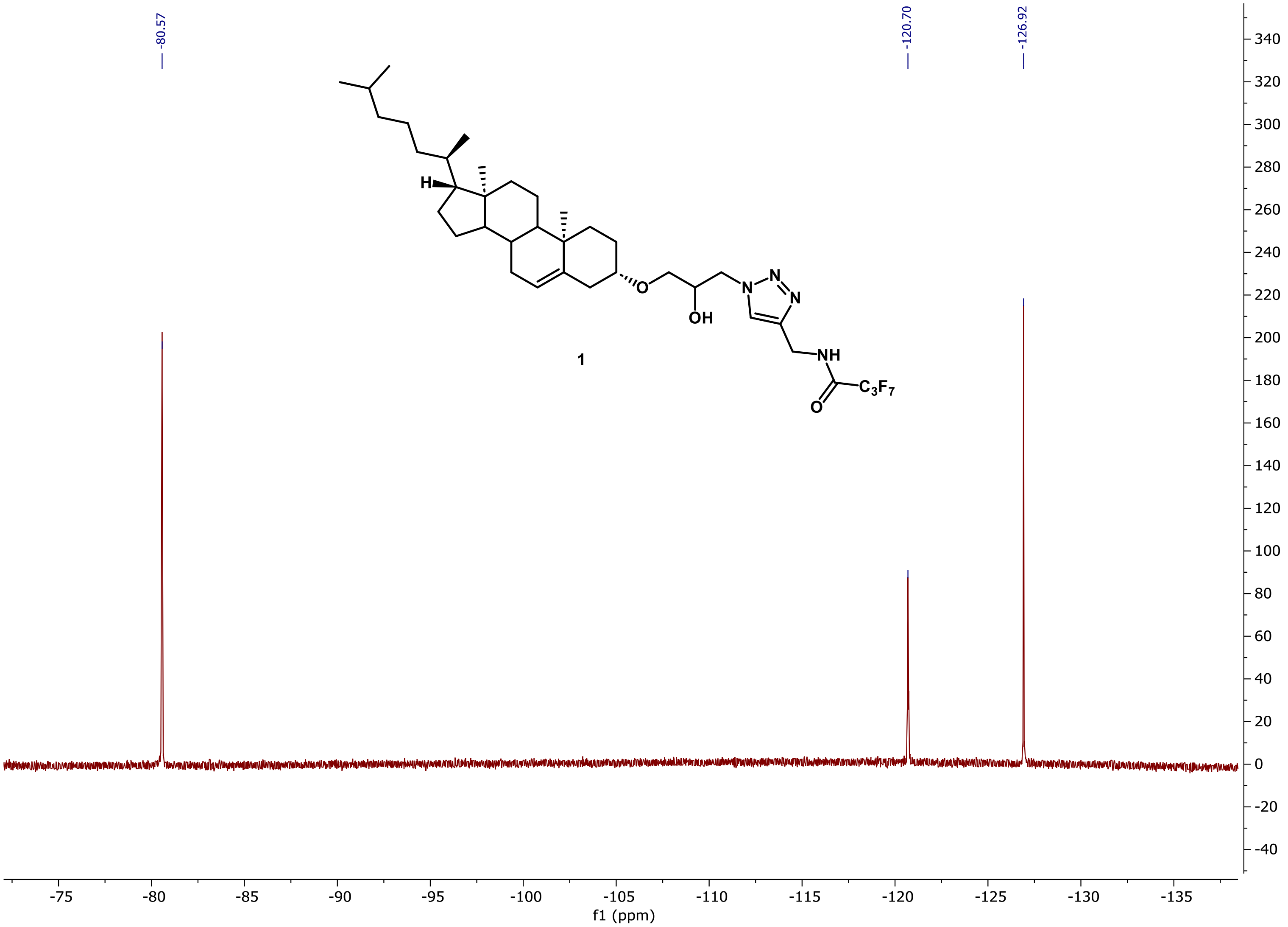

Figure S15. ^{19}F NMR spectrum of compound **1**.

*NMR spectra of compound **2***

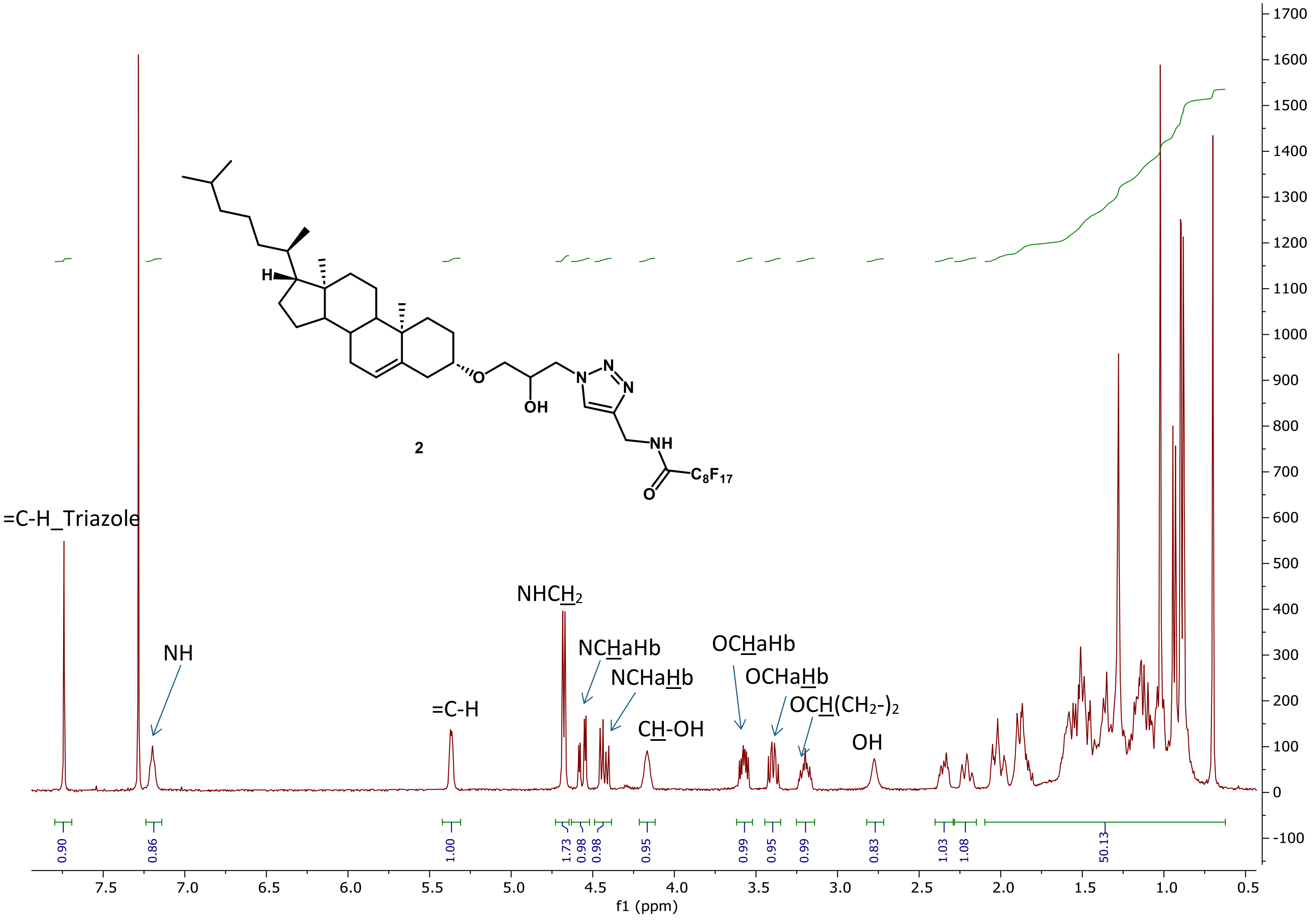

Figure S16. ^{1}H NMR spectrum of compound **2**.

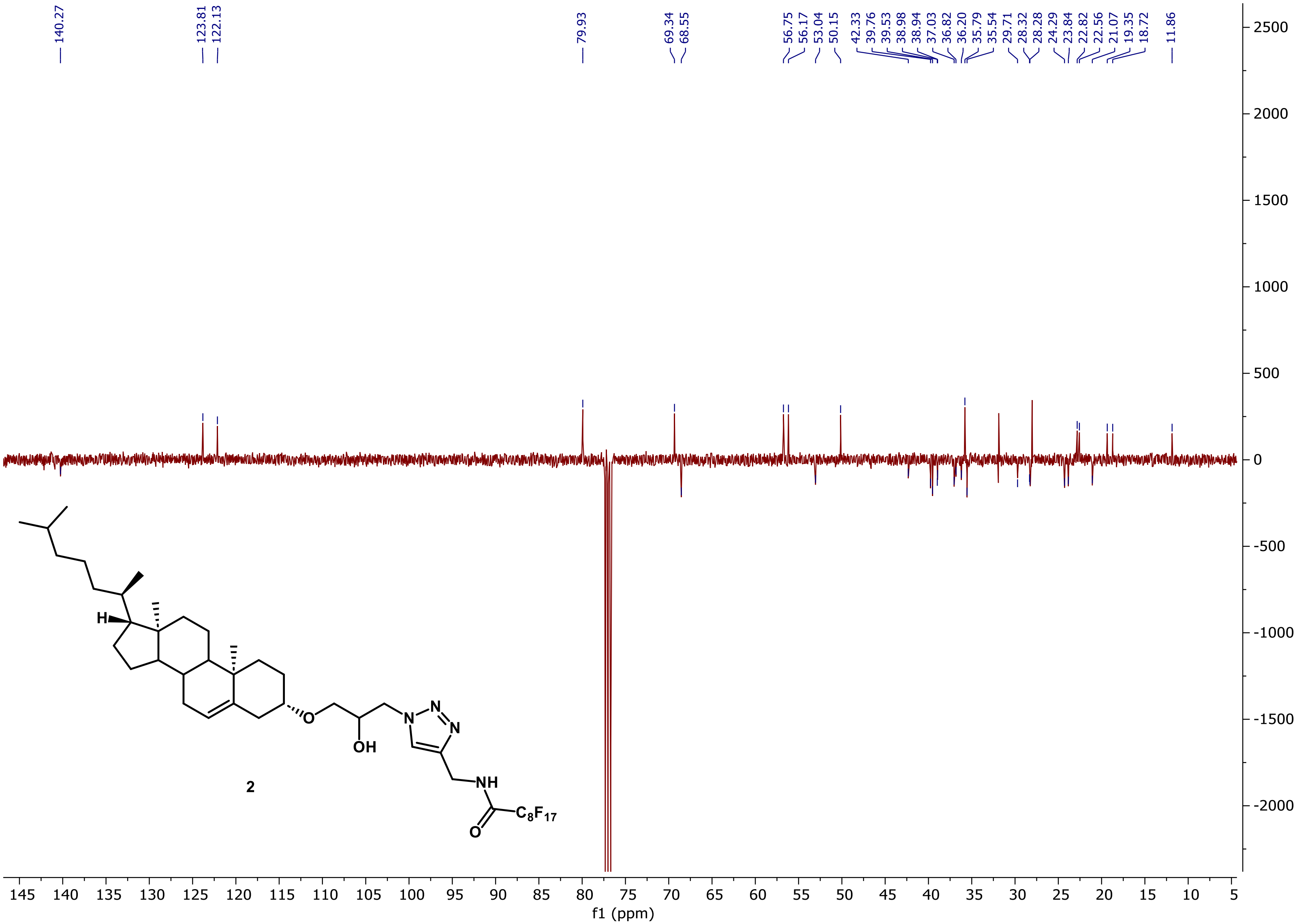

Figure S17. ^{13}C NMR spectrum of compound **2**.

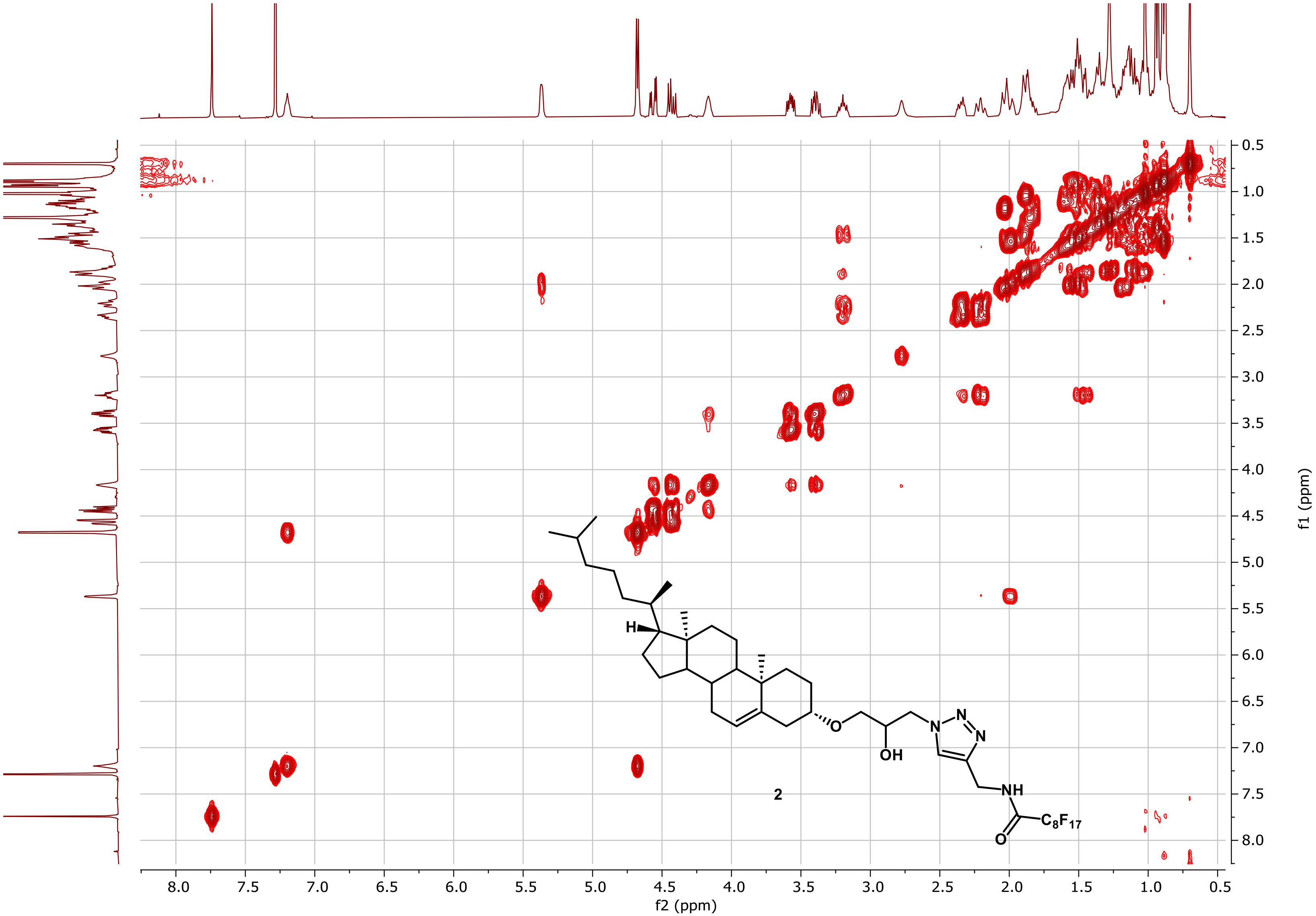

Figure S18. ^{1}H-^{1}H COSY NMR spectrum of compound **2**.

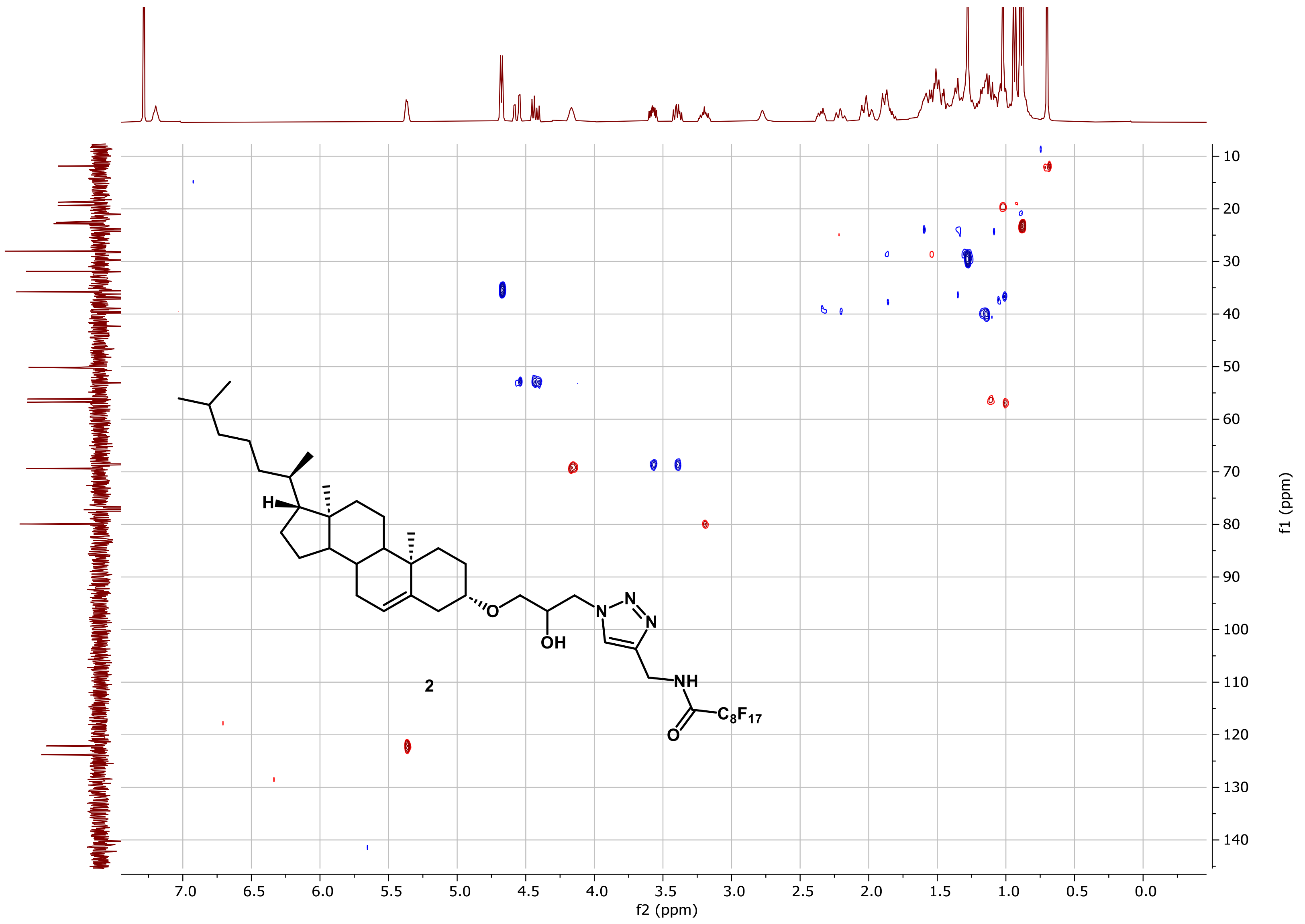

Figure S19. ^{1}H-^{13}C HSQC NMR spectrum of compound **2**.

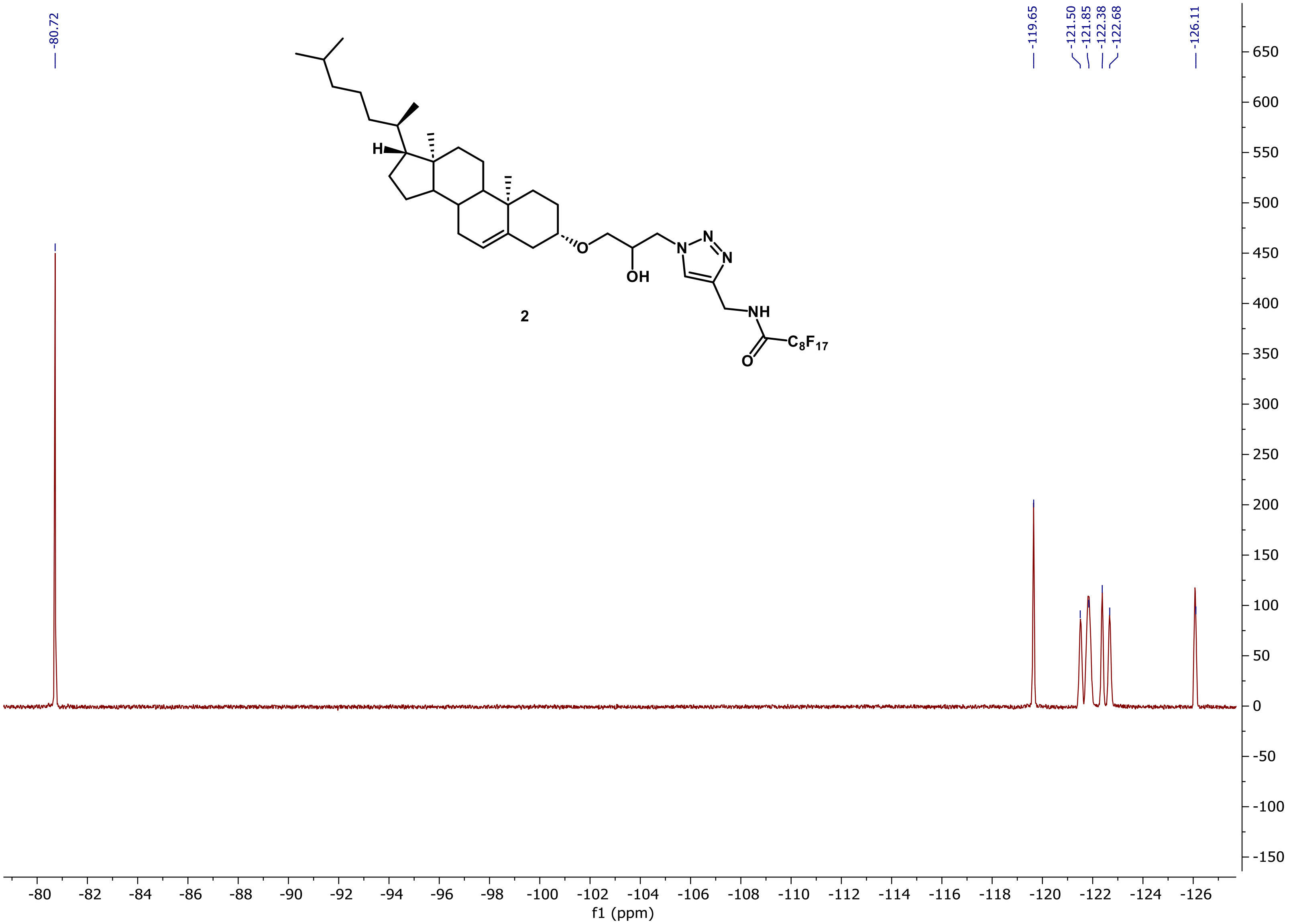

Figure S20. ^{19}F NMR spectrum of compound **2**.
